# Analysis of Floral Trait Diversity and Its Correlation with Fertility in *Hibiscus syriacus* Cultivars

**DOI:** 10.3390/plants15213492

**Published:** 2026-11-09

**Authors:** Zihao Mao, Yazhi Zhao, Fen Xiao, Chenyu Qi, Aoqing Bian, Xinyu Dou, Juyang Liao, Jianan Chen, Liqun Huang, Caixian Liu, Xiaohong Wang

**Affiliations:** 1 College of Landscape Architecture, Central South University of Forestry and Technology, Changsha 410004, China; 15675095169@163.com (Z.M.); 20241200545@csuft.edu.cn (Y.Z.); qcy2446254182@163.com (C.Q.); 19819110772@163.com (A.B.); a1945496048@126.com (X.D.); t20050193@csuft.edu.cn (L.H.); t20162284@csuft.edu.cn (C.L.); 2 Hunan Academy of Forestry, Changsha 410004, China; znl0718@163.com; 3 Hunan Botanical Garden, Changsha 410004, China; liaojuyang@163.com; 4 Ziyang District Forestry Bureau, Yiyang 413000, China; 13607370453@139.com; 5 Hunan Big Data Engineering Technology Research Center of Natural Protected Areas Landscape Resources, Changsha 410004, China

**Keywords:** *H. syriacus*, floral phenotype, fertility, pollen morphology, fruit-setting rate, correlation analysis

## Abstract

*Hibiscus syriacus* L. is a landscape plant with ornamental, economic, and ecological value. However, the low natural fruit-setting rate observed in most cultivars significantly hinders its crossbreeding, germplasm innovation, and large-scale application. We analyzed 20 cultivars to identify the key floral traits associated with *H. syriacus* fertility. Fifty floral phenotypic and pollen morphological indicators were systematically quantified, fruit-setting and pollen vitality rates were recorded, and insect foraging behaviors were observed to assess the diversity of floral characteristics and their correlation with fertility. The results showed that notable variations in floral phenotype and pollen morphology exist among the studied cultivars. Pollen grains were characterized by spinous ornamentation, with notable differences in grain size, spine morphology, and surface wrinkling. Correlation analysis identified nine core floral and palynological indicators that are highly associated with *H. syriacus* fertility. These comprise five floral phenotypic variables, such as pistil length (PL2) and heart length (HL), and four microscopic palynological features, including thorn curvature (TC) and spine number (TN). We built a phenotypic and palynological framework for analyzing fruit setting in *H. syriacus*. These nine key traits can act as morphological markers to quickly assess hybrid parents, supporting its genetic improvement, breeding and industrial promotion.

## 1. Introduction

*Hibiscus syriacus* L., commonly known as the rose of Sharon or hedge flower, is a deciduous shrub in the Malvaceae family [1]. This species is highly valued in landscaping for its diverse floral colors, extended flowering period, and strong stress resistance [2]. In addition to its ornamental appeal, it possesses significant edible and medicinal value [3], establishing it as an excellent greening plant for a cultivar of ecological applications [4]. Numerous cultivars of *H. syriacus* exist, displaying extensive diversity in floral phenotypes and palynological characteristics. However, most cultivars exhibit an extremely low natural fruit-setting rate [5], with obvious differentiation in fertility observed among cultivars; this represents a critical technical barrier that hinders germplasm innovation, limits crossbreeding efficiency, and restricts the large-scale industrial upgrading of the species. Recent research on *H. syriacus* has mostly focused on germplasm resource investigations, phenotypic diversity evaluations, and cultivation application. Although preliminary studies on fertility have been undertaken [6], some research has examined pollen morphology and fruiting separately. However, no study has systematically combined floral phenotype and pollen morphology in *H. syriacus* to identify which traits contribute to fertility. Studies on the plant fruit-setting rate and fertility have shown that floral phenotypes directly influence pollination efficiency and fertility [7]. Furthermore, pollen morphology is genetically stable, serving as an important indicator for taxonomic classification and the study of genetic relationships [8]. It is also closely linked to pollination and breeding outcomes [9,10,11], and it is correlated with pollen viability, stigma fertility, and gametophyte development [12,13,14]. These are the key factors related to the fruit-setting rate. Our team previously reported that the fertility of *H. syriacus* is closely associated with specific phenotypic traits and palynological characteristics [6], thereby providing an important basis for subsequent studies. Building upon previous research, this study has introduced new metrics, including field fruit-set rates and pollen viability statistics, conducted field surveys on pollinating insects, expanded the observation indicators for floral characteristics, and further refined the comprehensive evaluation system for *H. syriacus* fertility.

Despite the challenges of low fruit-setting rates and poor fertility in *H. syriacus*, a systematic correlation analysis of floral phenotype, pollen morphology, and fertility has yet to be conducted. This study aimed to clarify the relationship between fertility and related floral and pollen traits by investigating cultivars with divergent fruit-setting rates. By conducting this study, we identified the correlations between these traits and fertility, revealing the key factors that influence fruit-setting rates. These results provide technical support for the breeding of high-fertility *H. syriacus* cultivars and the sustainable development of the industry.

## 2. Results

### 2.1. Floral Phenotypic Traits of Cultivars

#### 2.1.1. Diversity of Floral Phenotypic Traits in *H. syriacus* Cultivars

The 20 tested *H. syriacus* cultivars exhibited rich diversity in quantitative and qualitative floral phenotypic traits.

Seventeen quantitative traits in the twenty cultivars showed considerable variation (Table S2). The CV ranged from 13.11% to 52.62%, with an average of 31.03%, in the following descending order: CW > HLL > PL1 > SN > SL > HL2 > HL > HL1 > BL > PW > SL1 > FD > CL > PL > PI > PL2 > BN. These results indicated that calyx width (CW, 52.62%) and heart line length (HLL, 51.85%) were prone to variation, whereas bract number (BN, 13.11%) was the most stable trait. The Shannon–Wiener diversity index (H′) of quantitative traits ranged from 1.23 to 1.98, with an average of 1.67, in the following descending order: SL1 > BL > PL > PW > PI > FD > PL2 > SL > PL1 > HLL > CL > HL1 > CW > SN > HL > HL2 > BN. Among these, stigma–style complex length (SL1, 1.98), bract length (BL, 1.96), and petal length (PL, 1.94) had the highest diversity indices, whereas the ratio of heart line length to heart length (HL2, 1.36) and bract number (BN, 1.23) had the lowest.

Qualitative floral traits exhibited significant variation among cultivars (Table S3), with flower color displaying up to six distinct morphotypes. The CV ranged from 22.80% to 75.29%, with a mean of 43.63%, in the following descending order: SC > STS > OP > SPD > HBS > FT > FC > RSB > OPP > PN > SS > FCN > RBH > BS > PWD > OS > HP > PF. Furthermore, the diversity index (H′) for these qualitative traits ranged from 0.19 to 1.67, with an average of 0.73, according to the descending order: FC > OP > SPD > FT > RBD > RSB > SS > BS > HBS > OPP > SC > STS > PN > PF > PWD > FCN > OS > HP. These results indicated that stigma color (SC, 75.29%), sepal tube shape (STS, 75.29%), and petal overlap (OP, 63.67%) possessed the highest CV values. Additionally, the highest diversity indices were observed for flower color (FC, 1.67) and petal overlap (OP, 1.29), and the lowest for ovary shape (OS, 0.33) and heart presence (HP, 0.20).

**Figure 1:**
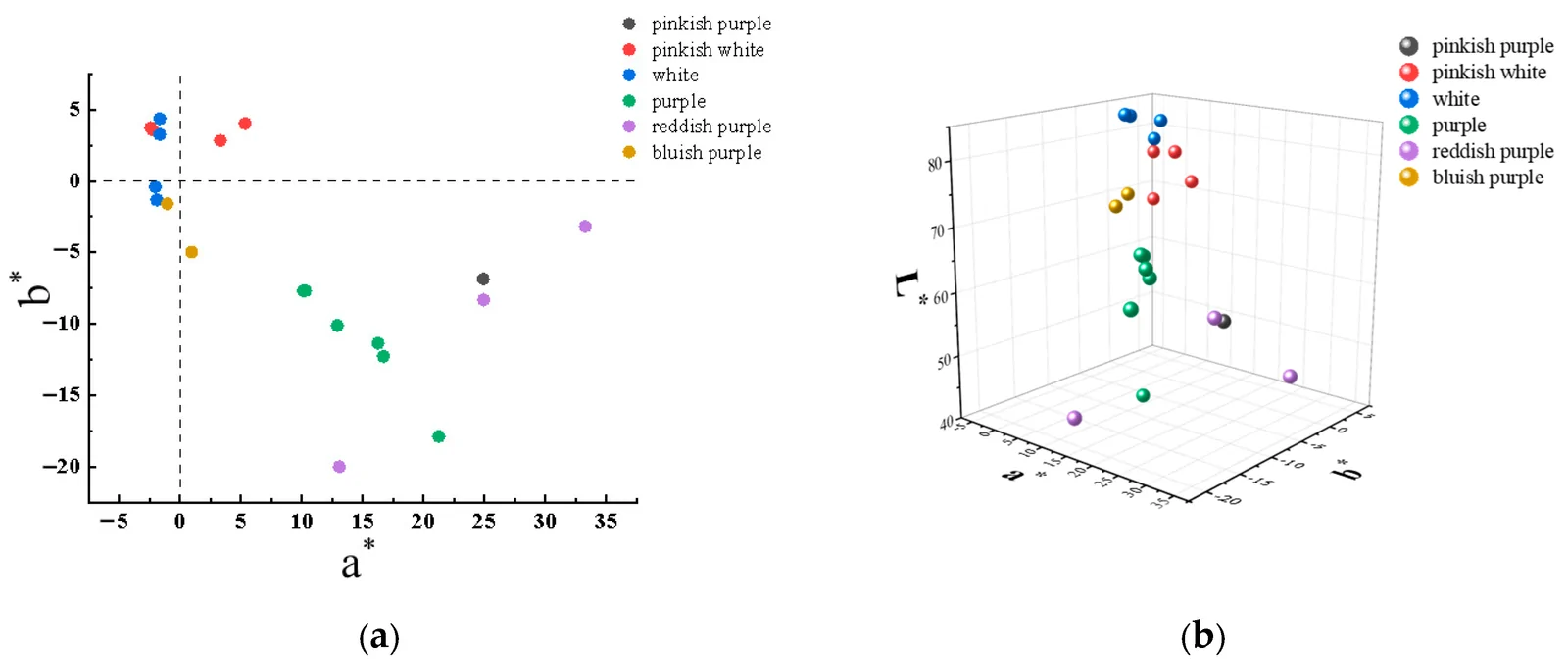
Flower color distribution of the *H. syriacus* cultivars. (**a**) Two-dimensional distribution map of a* and b* values. (**b**) Three-dimensional distribution map of L*, a*, and b* values.

Based on visual observation and color chart comparisons, the flower colors of the 20 cultivars were categorized into six distinct groups: white, pinkish-white, blue-purple, purple, pinkish-purple, and purplish-red. Based on colorimeter parameters, two-dimensional distribution maps of a* and b* values (Figure 1a) and three-dimensional distribution maps of L*, a*, and b* values (Figure 1b) were generated for each group. Within the two-dimensional a*–b* coordinate system, the color groups occupied four quadrants: pinkish-white was located primarily in the first and second quadrants, white was concentrated in the second and third quadrants, and the remaining color groups were situated mainly in the third and fourth quadrants, with white positioned near the origin. In the three-dimensional L*–a*–b* map, each category was clearly separated into concentrated clusters, suggesting that the six color groups defined by CIE Lab (a three-dimensional color coordinate system, L*: lightness; a*: red-green axis; b*: yellow-blue axis) were suitable for the classification of *H. syriacus* flower colors.

#### 2.1.2. Correlation and Cluster Analysis of Floral Phenotypes

**Figure 2:**
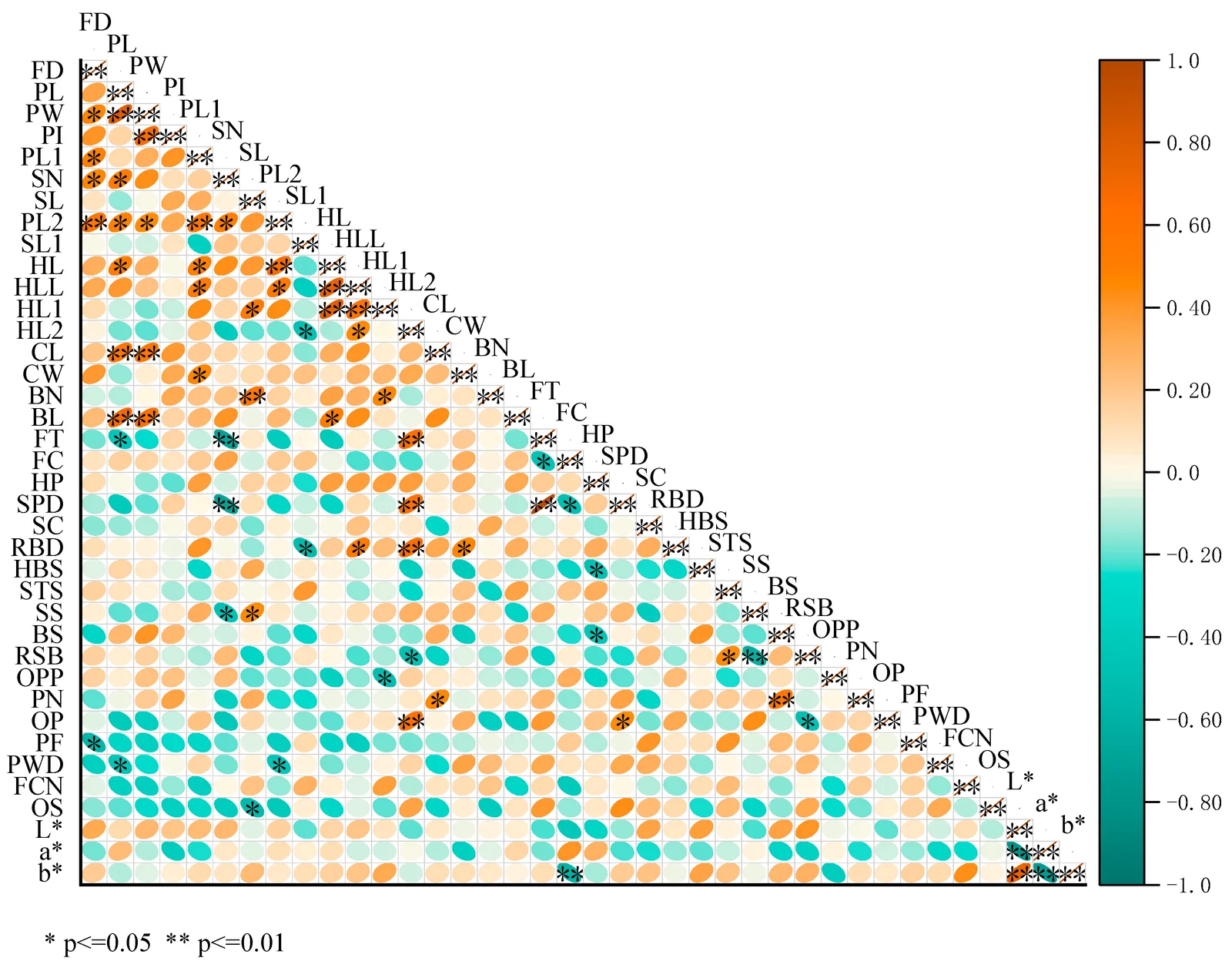
Correlation analysis for floral phenotypic traits in *H. syriacus*.

Heatmap analysis of the correlations among 38 traits in the 20 *H. syriacus* cultivars (Figure 2) demonstrated that quantitative traits were predominantly positively correlated, whereas qualitative traits were mainly negatively correlated.

Among the traits related to the heart line, heart line length (HLL) was significantly positively correlated with heart length (HL, 0.743**), heart length/petal length (HL1, 0.589**), and heart line length/heart length (HL2, 0.448*), together constituting a coordinately varied heart characteristic system. Regarding pistil-length-related traits, pistil length (PL2) was significantly positively correlated with flower diameter (FD, 0.582**), pedicel length (PL1, 0.577**), and heart line length (HLL, 0.558*), indicating that pistil development was closely associated with overall flower size and heart traits. For flower-type-associated traits, flower type (FT) and stamen petalization degree (SPD, 0.948**) were significantly correlated with petal length (PL, −0.452*). These results suggest that a higher degree of doubling was associated with reduced petal length and stamen development. Meanwhile, FC, a qualitative trait, was significantly correlated with color parameters such as the yellow–blue value (b*, −0.592**).

**Figure 3:**
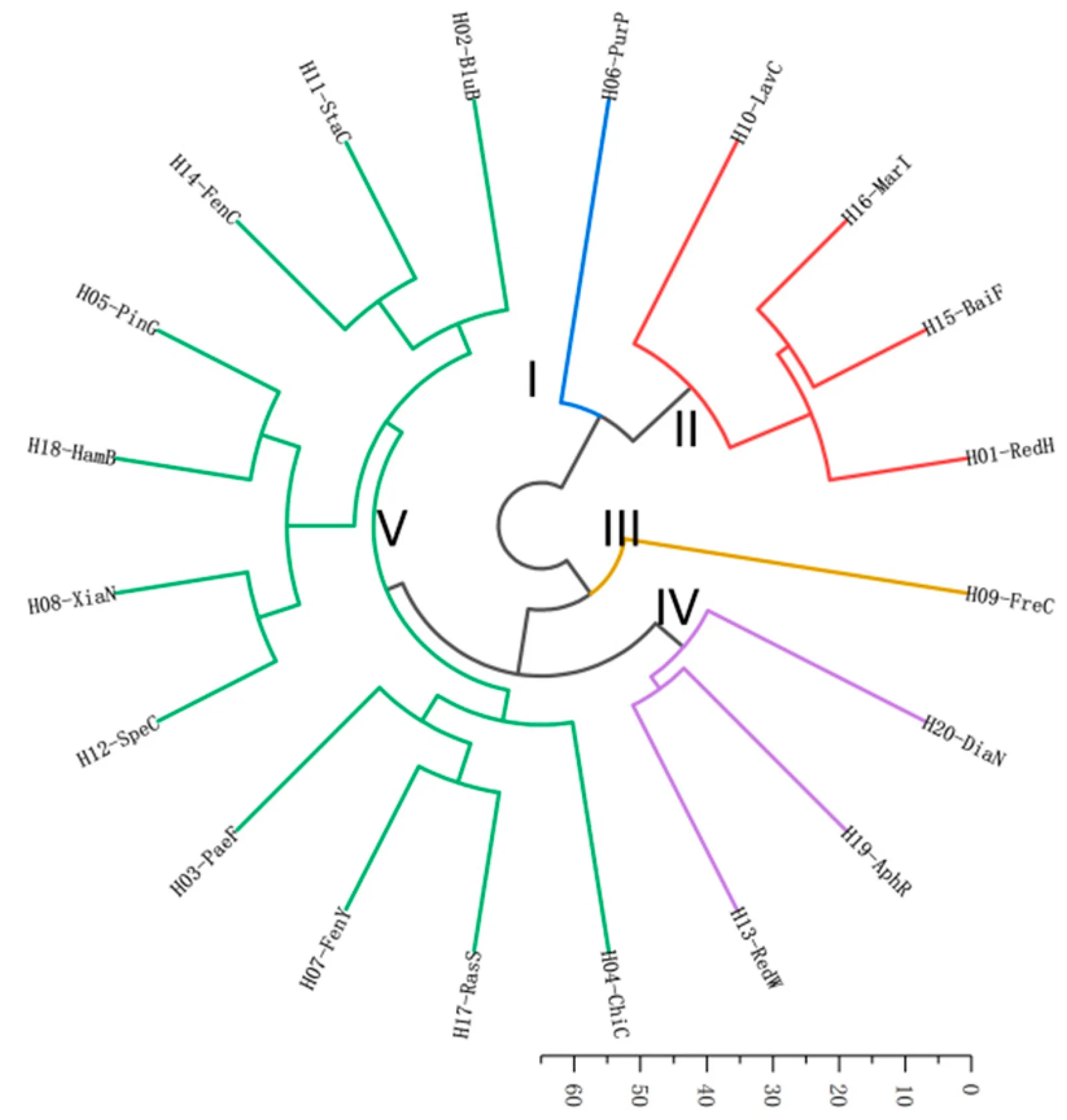
Q-type cluster analysis based on 38 floral phenotypic traits of *H. syriacus*.

Based on the cluster analysis of floral phenotypic traits, the 20 *H. syriacus* cultivars were categorized into five distinct groups (Figure 3). Group I consisted of one accession (5.00%; H06-PurP), characterized by a heart length (HL) and heart line length (HLL) significantly greater than those of other cultivars. Group II comprised four cultivars (20.00%; H10-LavC, H16-MarI, H15-BaiF, H01-RedH), which exhibited a relatively large flower diameter (FD) and petal index (PI). Group III included one accession (5.00%; H09-FreC) with a high degree of doubling. Group IV contained three cultivars (15.00%; H20-DiaN, H19-AphR, H13-RedW), which were distinguished by a short pedicel length (PL1). The remaining 11 cultivars were classified into Group V (55.00%), characterized by relatively short pistil length (PL2).

### 2.2. H. syriacus Pollen Morphology

#### 2.2.1. Analysis of Pollen Morphological Diversity in *H. syriacus*

Pollen grains across all cultivars were monads, subspherical, and characterized by spinose ornamentation. The spines were conical with obtuse apices, and scattered pores were evenly distributed around the microspines (Figure S1). Granular protuberances were also observed on the pollen surface (photo 5 and photo 7 in Figure S2).

**Figure 4:**
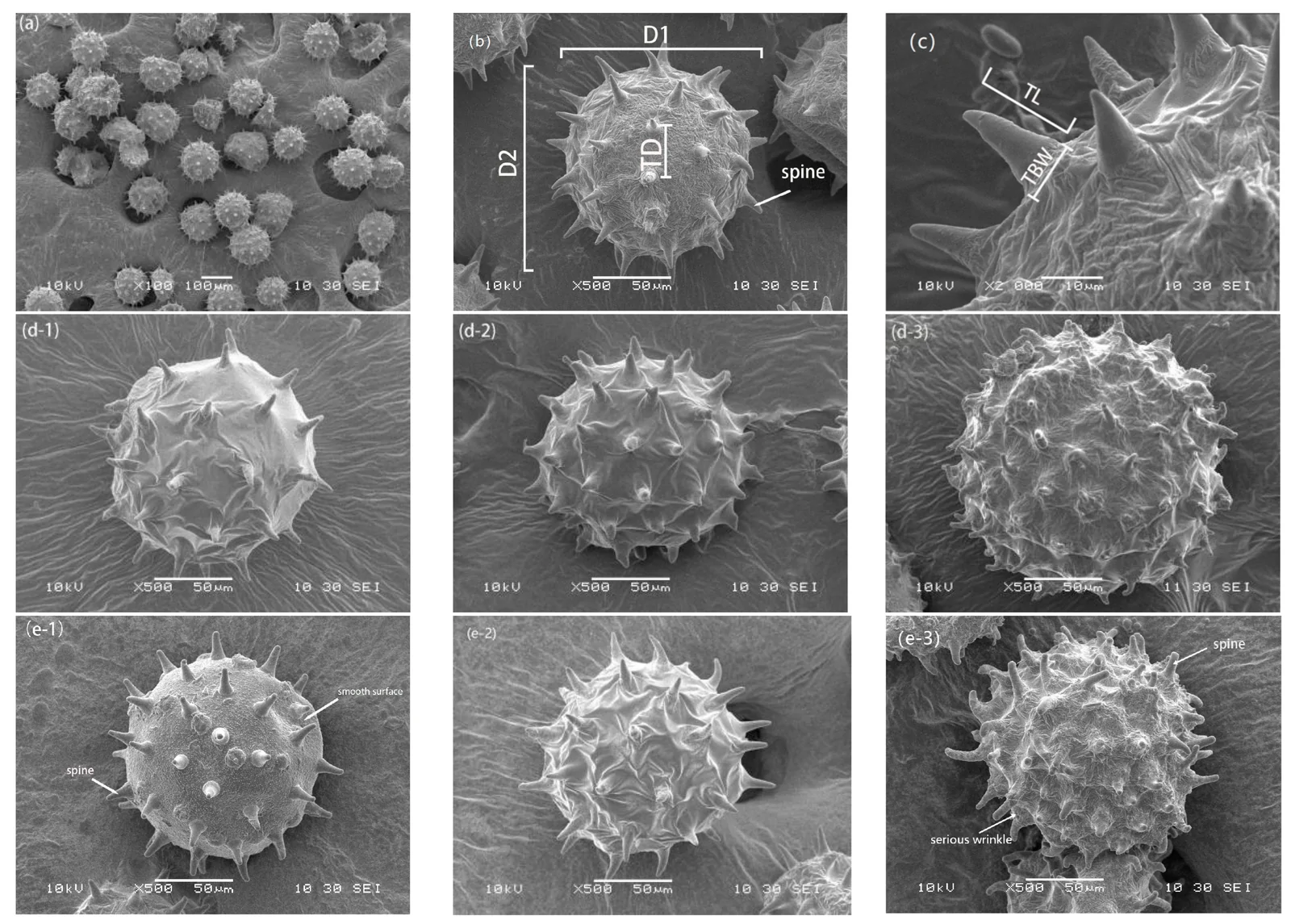

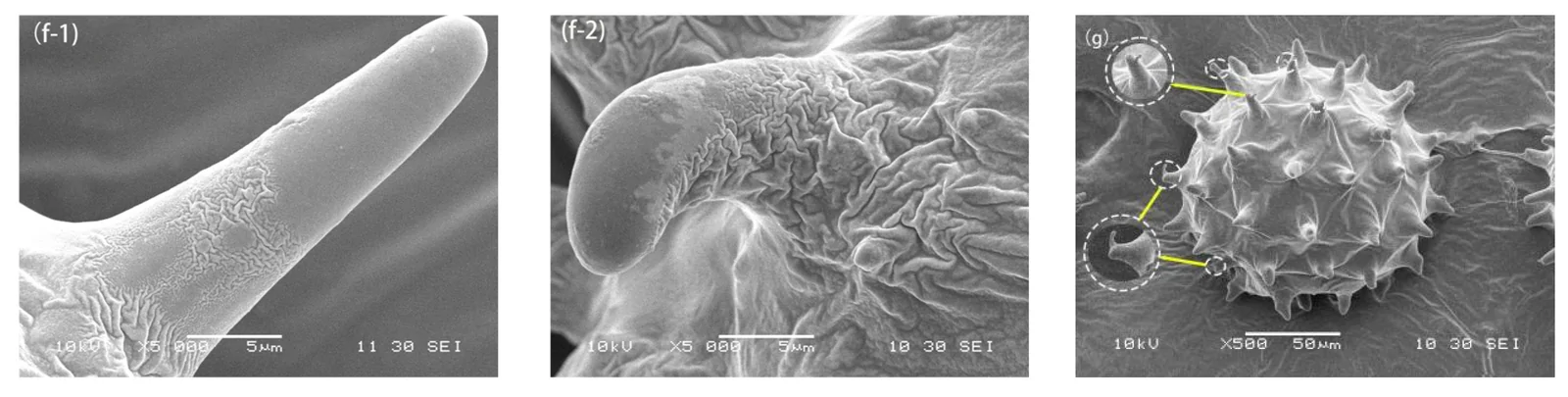
Scanning electron microscopic images of *H. syriacus* pollen grain morphology. (**a**) Spiny spherical pollen; (**b**) Pollen transverse diameters, pollen longitudinal diameters, and spine interval. (**c**) Pollen spine length and base width of spines. The number of spines in a frontal view of a grain: (**d-1**) below 30; (**d-2**) ranging from 30 to 40; (**d-3**) exceeding 40. (**e-1**) Smooth pollen surface; (**e-2**) moderately wrinkled pollen surface; (**e-3**) severely wrinkled pollen surface. (**f-1**) Erect pollen spines; (**f-2**) curved pollen spines. (**g**) Microspines on the pollen spine surface.

Detailed data regarding qualitative pollen traits are presented in Table S4. Pollen shape was categorized as subspherical (80.00%) or spherical (20.00%). Surface wrinkling varied significantly among cultivars, with most appearing wrinkled and only a few identified as smooth. Furthermore, pollen spines were classified into straight or curved types, with some cultivars possessing microspines (Figure S2, Figure 4).

**Figure 5:**
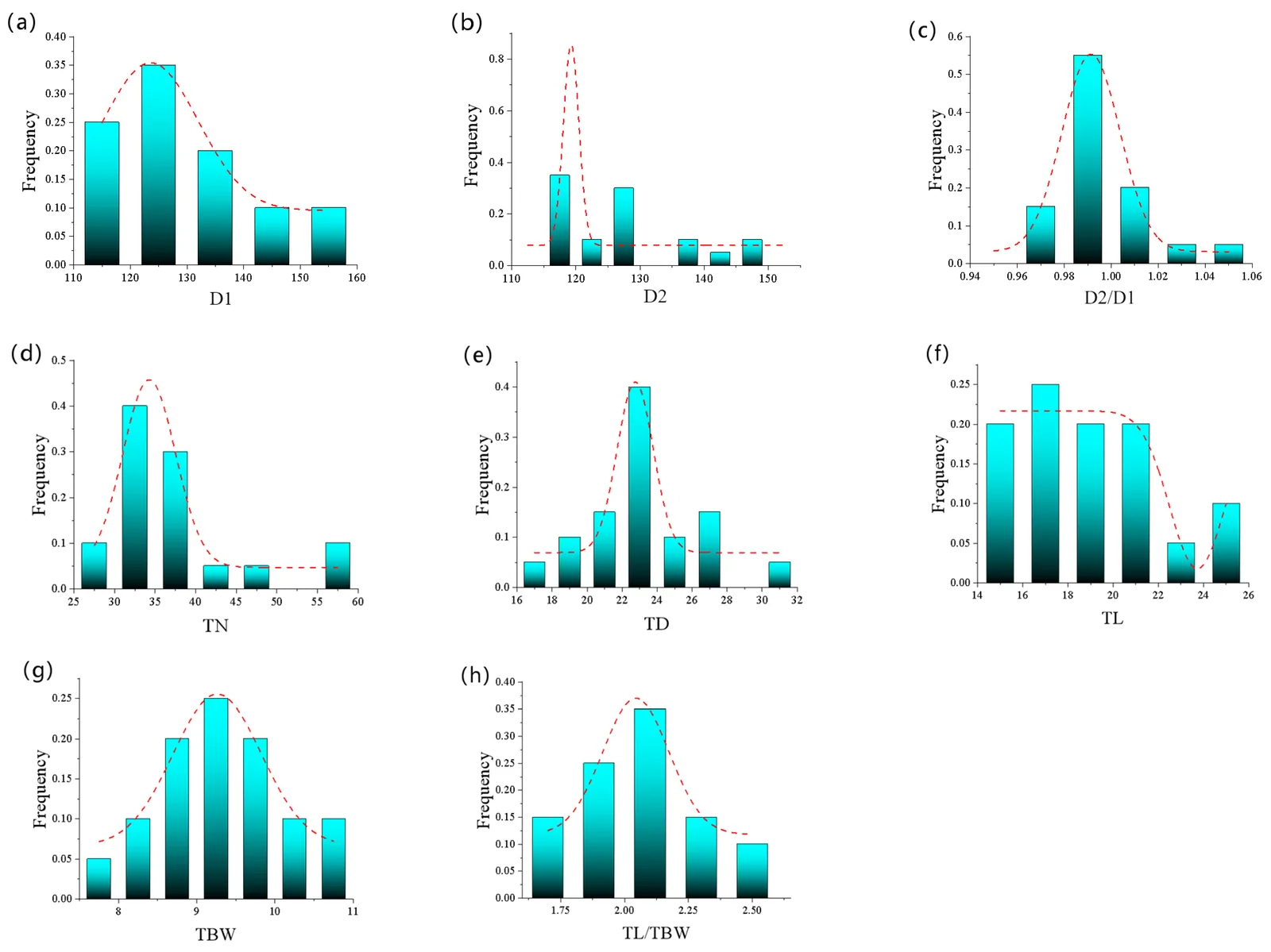
Bar chart of quantitative pollen trait distribution in 20 *H. syriacus* cultivars. (**a**) pollen transverse diameters (D1; range: 115.52–154.75 µm, peak: 125–135 µm). (**b**) pollen longitudinal diameters (D2; range: 115.52–149.74 µm, peak: 125–135 µm). (**c**) D2/D1 ratio (range: 0.96–1.04, peak: 0.98–1.00). (**d**) Spine number (TN; range: 28–58, peak: 35–40). (**e**) Spine distance (TD; range: 17.5–31.04 µm, peak: 22–24 µm). (**f**) Spine length (TL; range: 15.39–24.75 µm, peak: 18–20 µm). (**g**) Spine basal width (TBW; range: 7.81–10.92 µm, peak: 9.0–9.5 µm). (**h**) TL/TBW (range: 1.68–2.46, peak: 2.0–2.2).

Quantitative pollen traits of *H. syriacus* are detailed in Table S4 and Figure 5. The pollen transverse diameters (D1) averaged 129.40 μm (range: 115.52–154.75 μm), whereas the pollen longitudinal diameters (D2) averaged 128.01 μm (range: 115.52–149.74 μm). The D2/D1 ratio averaged 0.99 (range: 0.96–1.04), displaying a low CV; the maximum D2/D1 ratio was observed in ‘Bai Fengche’ and the minimum in ‘Blue Bird’.

The mean spine number (TN) for *H. syriacus* pollen was 36.2 (range: 28–58), with differences noted among cultivars. Although most cultivars exhibited a relatively low TN, ‘Red Woodbridge,’ ‘Raspberry Smoothie,’ and ‘Pink Giant’ displayed elevated counts. The mean spine distance (TD) was 23.12 μm (range: 17.5–31.04 μm), with ‘China Chiffon’ and ‘Raspberry Smoothie’ exhibiting the maximum and minimum values, respectively.

The mean spine length (TL) of the 20 cultivars was 19.38 μm (range: 15.39–24.75 μm). Variation was observed among varieties, with ‘Diana’ exhibiting the maximum TL and ‘Marina’ the minimum. The mean spine basal width (TBW) was 9.34 μm (range: 7.81–10.92 μm); ‘French Cabaret Purple’ had the largest TBW, whereas ‘Pink Giant’ had the smallest. Finally, the mean TL/TBW ratio was 2.08 (range: 1.68–2.46), with the highest ratio found in ‘Diana’ and the lowest in ‘Blue Bird.’

#### 2.2.2. Correlation and Cluster Analysis of Pollen Morphology in *H. syriacus*

**Figure 6:**
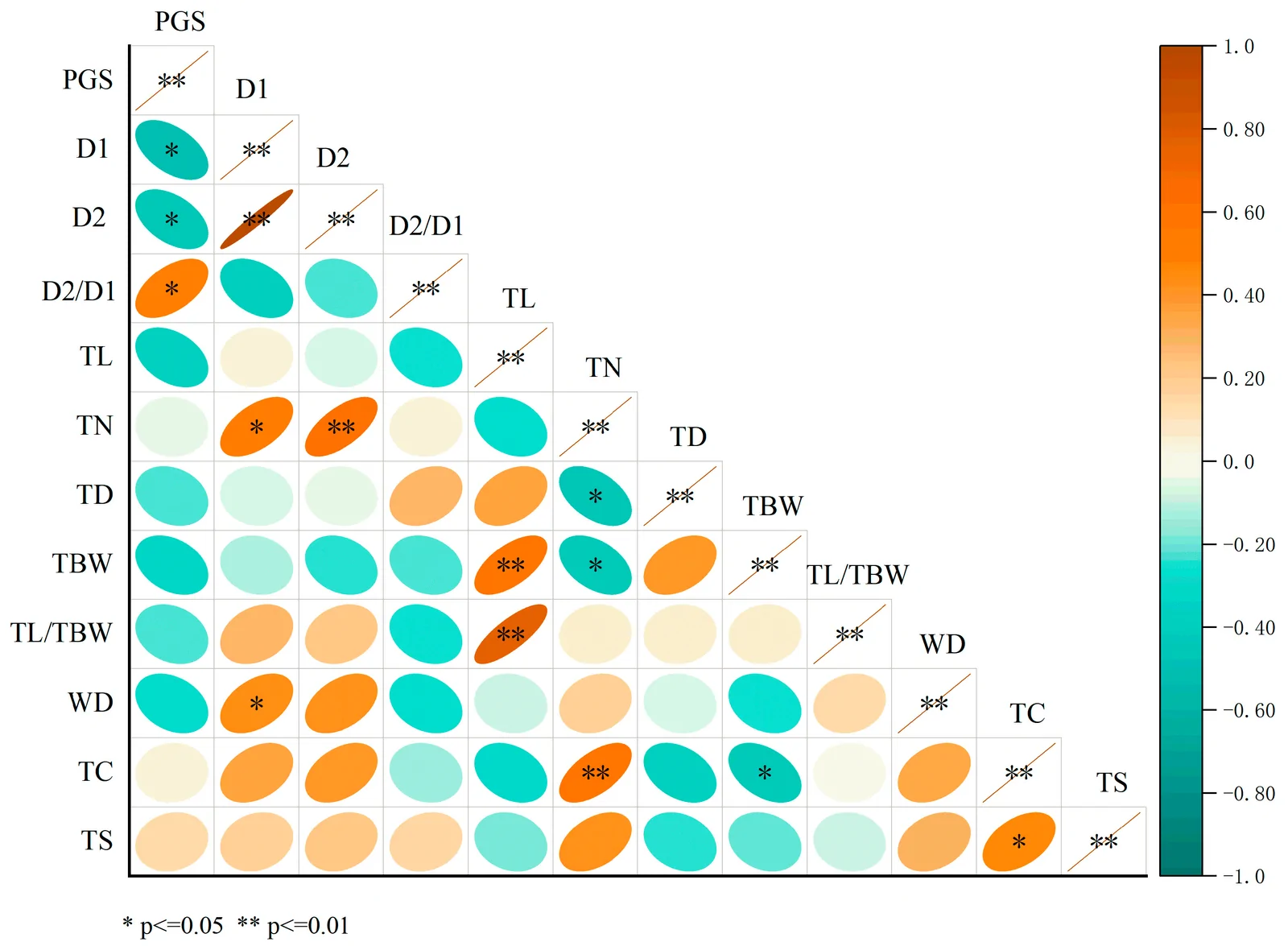
Correlation analysis of pollen morphological traits in *H. syriacus*.

Heatmap analysis of significant correlations among 12 pollen morphological traits across 20 *H. syriacus* cultivars (Figure 6) revealed four groups of positively correlated variables. Positive correlations were observed among D1, pollen longitudinal diameters (D2), spine number, and wrinkle degree (WD); among spine length (TL) and the TL/TBW ratio (0.802**); among spine curvature (TC) and TN (0.605**); and, finally, between TC and TS (0.464*). Three groups of negatively correlated variables were also identified. The first included pollen grain shape (PGS) and D1 (−0.520*); in the second group, TN was correlated with TD (−0.488*) and TBW (−0.45*); and the third involved TBW and TC (−0.444*).

These results indicated that the pollen traits of *H. syriacus* do not vary independently, and that significant synergistic or antagonistic correlations exist among pollen size, exine ornamentation, surface microstructure, and other morphological characteristics.

**Figure 7:**
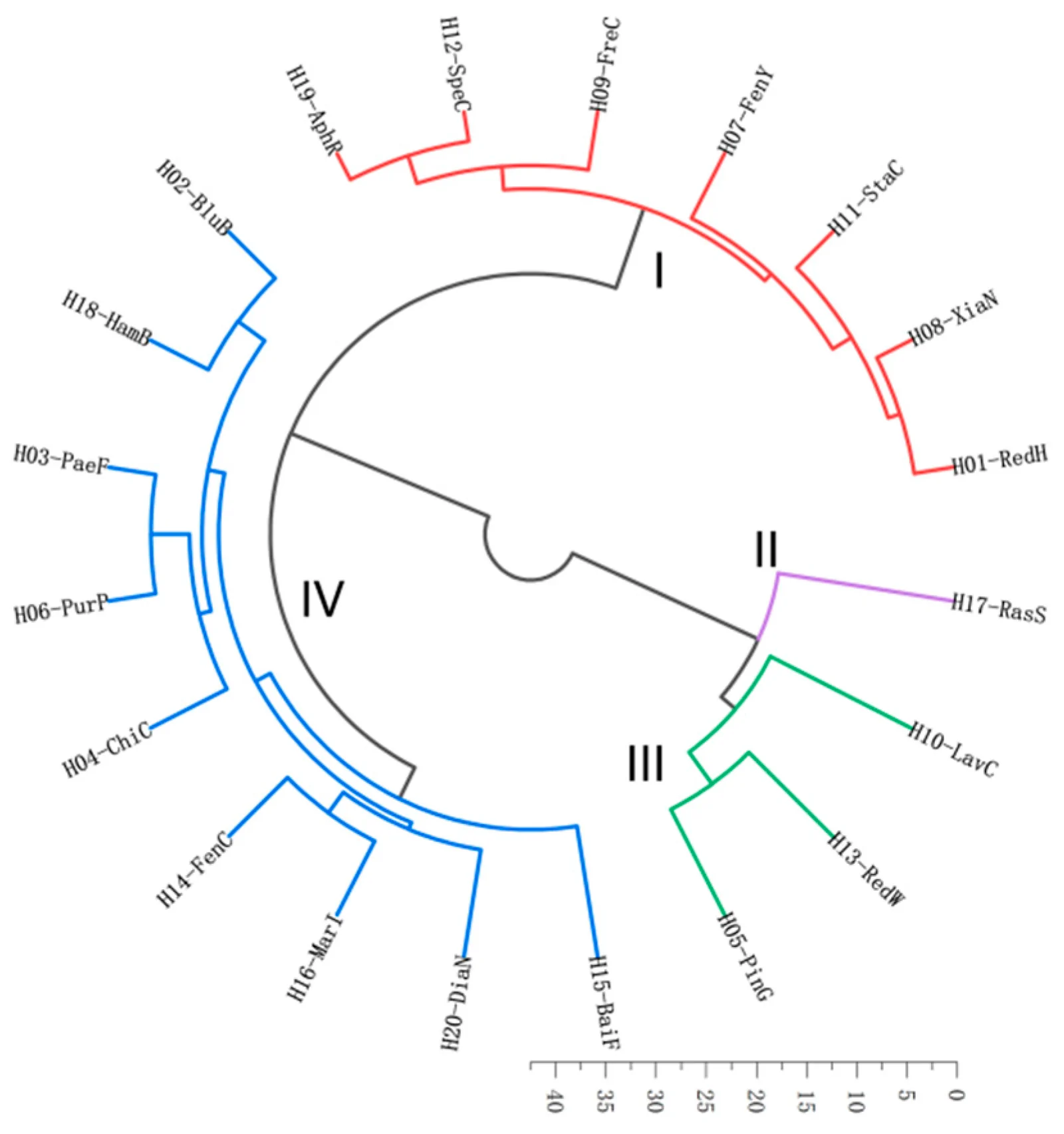
Q-type cluster analysis based on 12 pollen morphological traits of *H. syriacus*.

Based on a cluster analysis of 12 palynological traits, the 20 *H. syriacus* cultivars were categorized into four distinct groups (Figure 7). Group I comprised seven cultivars (35.00%; H19-AphR, H12-SpeC, H09-FreC, H07-FenY, H11-StaC, H08-XiaN, and H01-RedH), characterized by pollen transverse diameter (D1) and longitudinal diameters (D2) below 120 μm. Group II consisted of a single accession (5.00%; H17-RasS) distinguished by a narrow spine distance (TD). Group III included three cultivars (15.00%; H10-LavC, H13-RedW, and H05-PinG), defined by D1 and D2 measurements exceeding 140 μm and a high spine number (TN). Finally, Group IV contained nine cultivars (45.00%; H02-BluB, H18-HamB, H03-PaeF, H06-PurP, H04-ChiC, H14-FenC, H16-MarI, H20-DiaN, and H15-BaiF) with D1 and D2 values ranging from 120 μm to 140 μm.

### 2.3. Fertility Insect Flower-Visiting Behavior for H. syriacus Cultivars

#### 2.3.1. *H. syriacus* Cultivar Fertility

**Figure 8:**
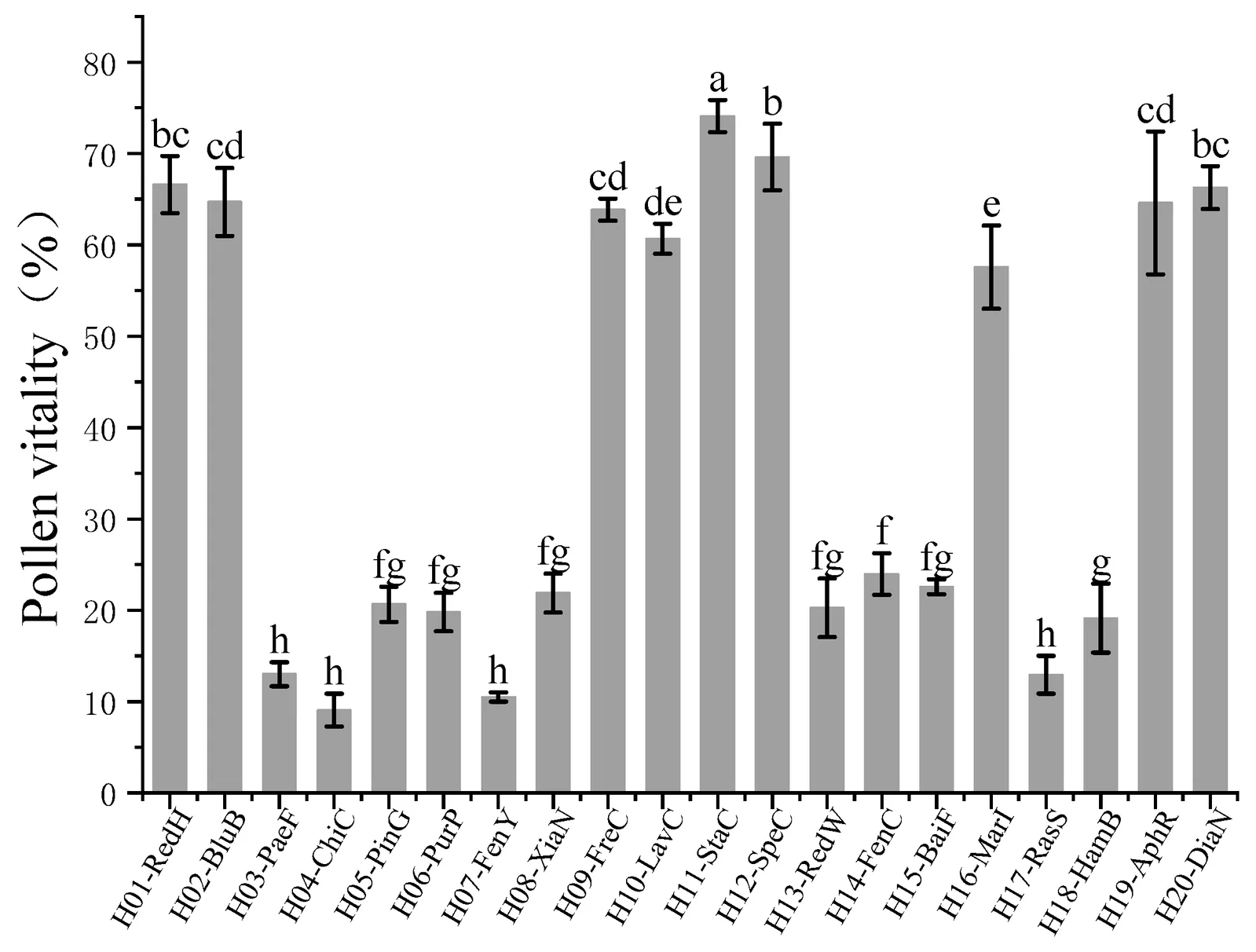
Differences in pollen viability across different *H. syriacus* varieties. Note: Different letters above bars represent differences among groups.

Pollen viability differed among the 20 *H. syriacus* cultivars. Overall, single-petaled cultivars exhibited notably higher pollen viability than semi-double-petaled cultivars, whereas semi-double-petaled cultivars exhibited higher pollen viability than double-petaled cultivars. Regarding fertility, the cultivars ranked in the following order: ‘Starlight Chiffon’ > ‘Wild-type Species’ > ‘Red Heart’ > ‘Diana’ > ‘Blue Bird’ > ‘Aphrodite’ > ‘French Cabaret Purple’ > ‘Lavender Chiffon’ > ‘Marina’ > ‘Fen Chiffon’ > ‘Bai Fengche’ > ‘Xiao Nvhai’ > ‘Pink Giant’ > ‘Red Woodbridge’ > ‘Purple Pillar’ > ‘Hamabo’ > ‘Paeonyflorus’ > ‘Raspberry Smoothie’ > ‘Fen Yun’ > ‘China Chiffon’ (Figure 8).

Fertility grades were classified based on fruit-setting rates under open and artificial pollination conditions, as determined from their three consecutive years of field observations. The fruit-set rate under artificial pollination served as the primary evaluation criterion. The results showed that there was significant fertility differentiation among the 20 *H. syriacus* cultivars, with 13 low-fertility cultivars, 3 medium-fertility cultivars, and 4 high-fertility cultivars identified (Table 1).

#### 2.3.2. Insect Flower Visitation

**Figure 9:**
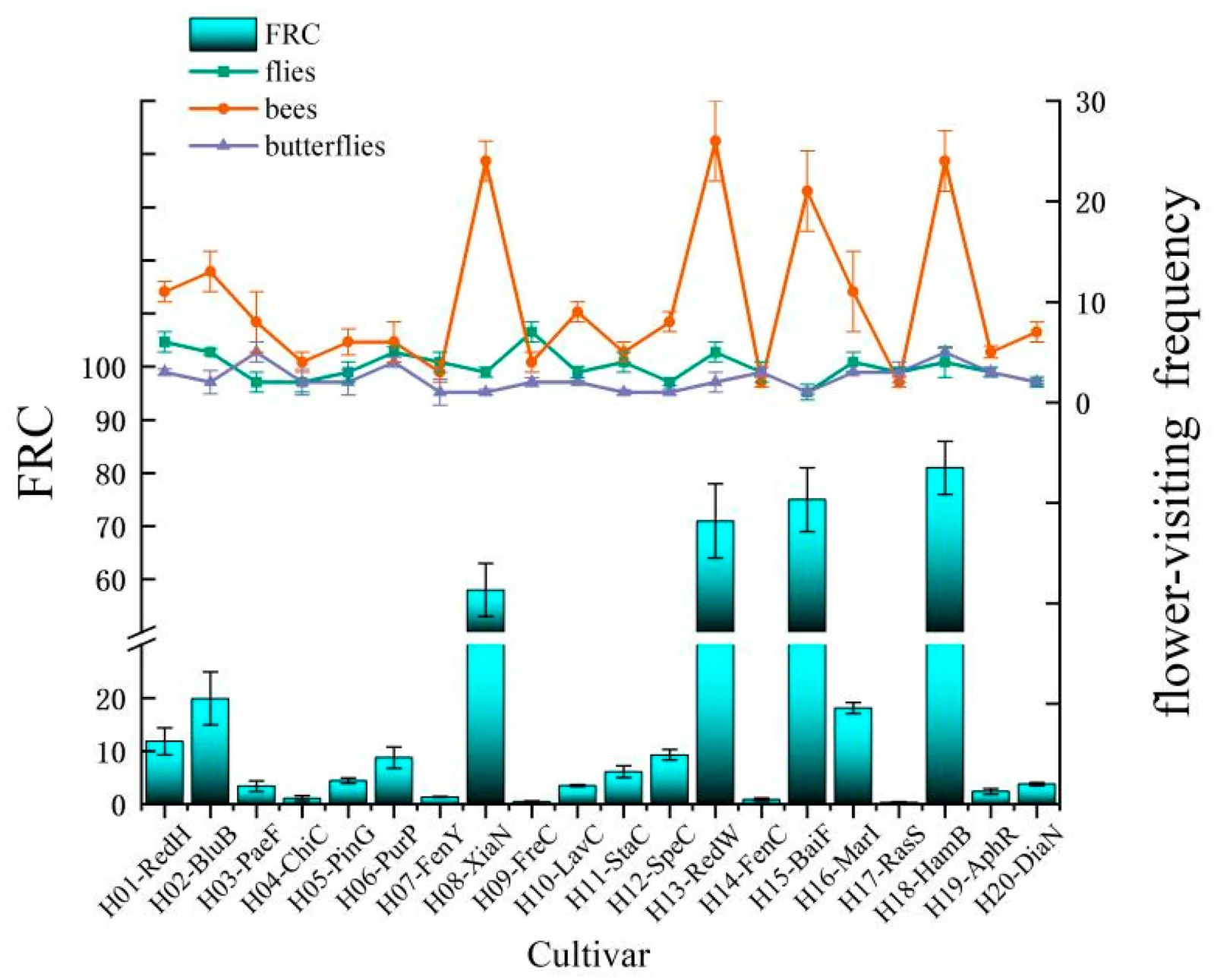
Dual-*Y*-axis column and line chart of the fruit-setting rate and insect flower-visiting frequency in 20 *H. syriacus* cultivars.

Field observations showed that the main flower-visiting insects of *H. syriacus* were flies, bees, and butterflies, with the overall visitation frequency of bees > flies > butterflies, displaying bee visitation peaks in some cultivars. There were differences (Figure 9) in insect visitation frequencies among different *H. syriacus* cultivars, and cultivars with higher fruit-setting rates corresponded to higher flower-visiting frequencies, particularly for bees (Figure 9).

### 2.4. Correlations Between Floral Phenotype and Pollen Morphological Traits with Fertility in H. syriacus

In this study, we employed correlation coefficient analysis via SPSS 25.0 to identify nine indicators significantly associated with fertility in *H. syriacus*, encompassing floral phenotype and pollen morphology (Table S5).

#### 2.4.1. Correlation Analysis Between Floral Phenotype and Fertility

**Figure 10:**
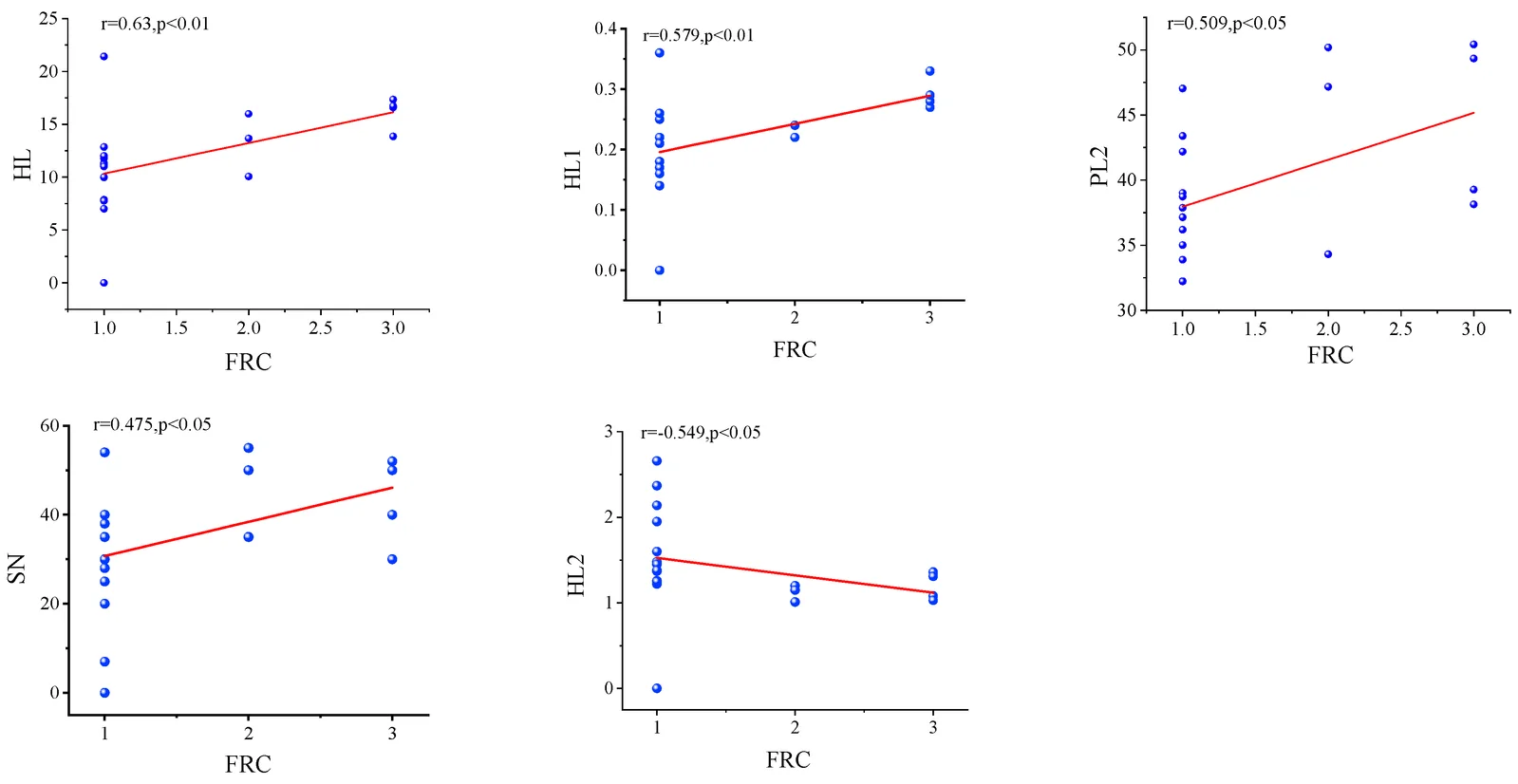
Correlation analysis of floral phenotype traits and fertility in *H. syriacus*.

Floral phenotypic traits were divided into quantitative and qualitative traits, and Spearman correlation analyses were conducted separately to assess the association between each trait category and fertility. After FDR correction, five quantitative floral traits were found to be significantly correlated with fertility (Figure 10), while no significant correlations were observed for qualitative floral traits. Among them, heart length (HL, r = 0.63**), the ratio of heart length to petal length (HL1, r = 0.579**), pistil length (PL2, r = 0.509*) and stamen number (SN, r = 0.475*) showed significant positive correlations with fertility. The ratio of heart line length to heart length (HL2, r = −0.549*) was significantly negatively correlated with fertility.

#### 2.4.2. Correlation Analysis for Pollen Morphology and Fertility

**Figure 11:**
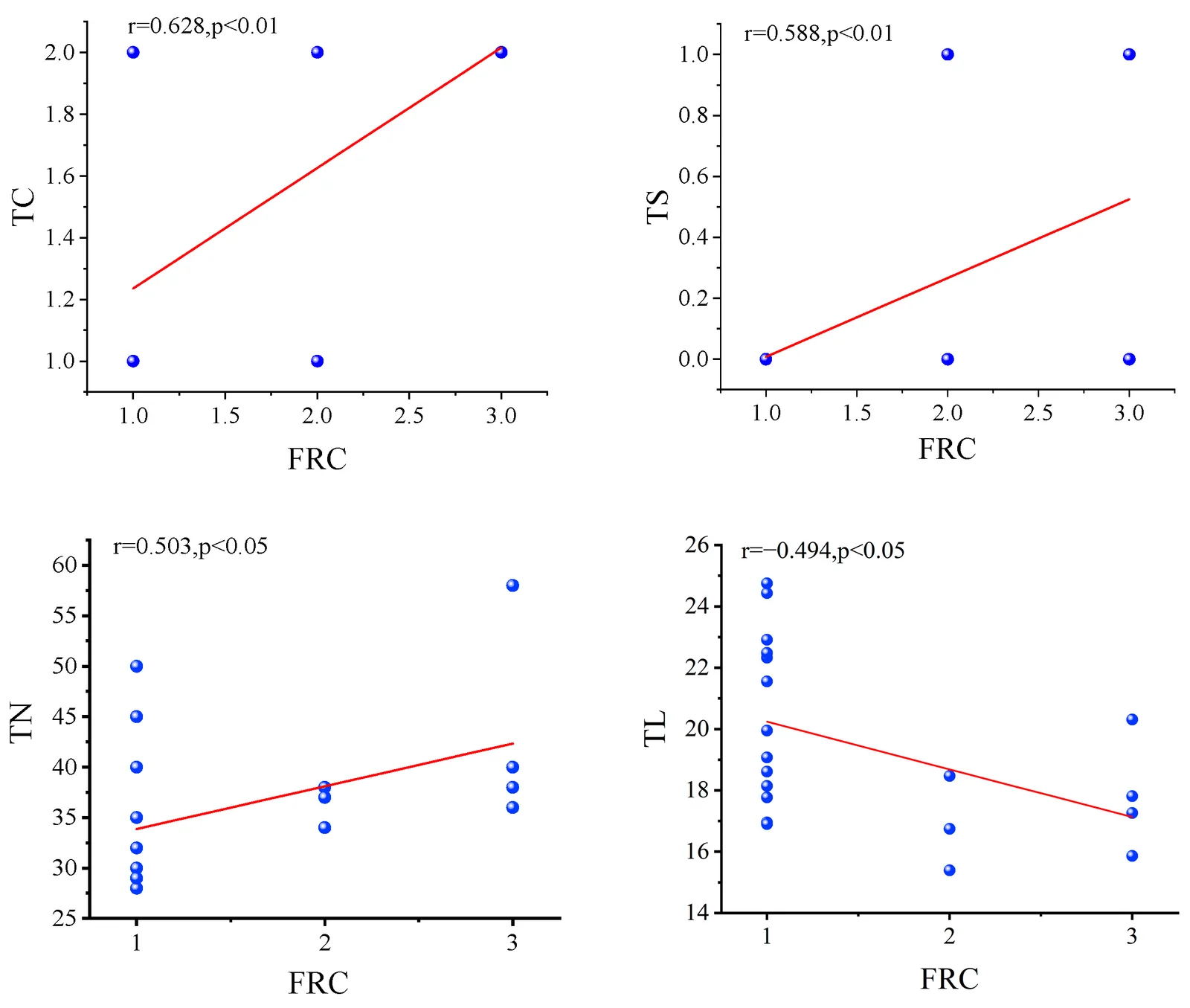
Correlation analysis of pollen morphological traits and fertility in *H. syriacus*.

A Spearman correlation analysis was conducted for 12 pollen morphological indicators and fertility. A total of four pollen morphological traits showed significant or extremely significant correlations with *H. syriacus* fertility (Figure 11). Among them, spine curvature (TC, r = 0.628**), spine surface (TS, r = 0.588**) and spine number (TN, r = 0.503*) were significantly positively correlated with fertility, whereas spine length (TL, r = −0.494*) was negatively correlated with fertility.

#### 2.4.3. Principal Component Analysis of Pollen Morphological and Floral Phenotypic Traits

The correlation analysis of pollen morphological and floral phenotypic traits (Table S6) identified 19 fertility-related indicators for subsequent principal component (PCA) and comprehensive clustering analyses of the 20 *H. syriacus* cultivars. PCA was used to reduce the data dimensions to identify key fertility-related factors, whereas cluster analysis grouped the cultivars to reveal differences in fertility-related phenotypic characteristics.

KMO and Bartlett’s test of sphericity were assessed before conducting the PCA. The indices met the required criteria (KMO ≥ 0.6, *p* < 0.01) [15], indicating that the dataset was suitable for PCA.

The floral phenotypic indicators consisted of 13 parameters: pedicel length (PL1), pistil length (PL2), outer petal posture (OPP), colorimetric value L*, heart presence (HP), sepal tube shape (STS), petal form (PF), stamen number (SN), stigma–style complex length (SL1), flower type (FT), stamen petaloid degree (SPD), heart length (HL), and the ratio of heart length to petal length (HL1). For pollen morphology, the indicators included six parameters: the ratio of pollen longitudinal diameters to pollen transverse diameters (D2/D1), spine length (TL), spine number (TN), spine surface (TS), spine curvature (TC), and wrinkle degree (WD).

**Figure 12:**
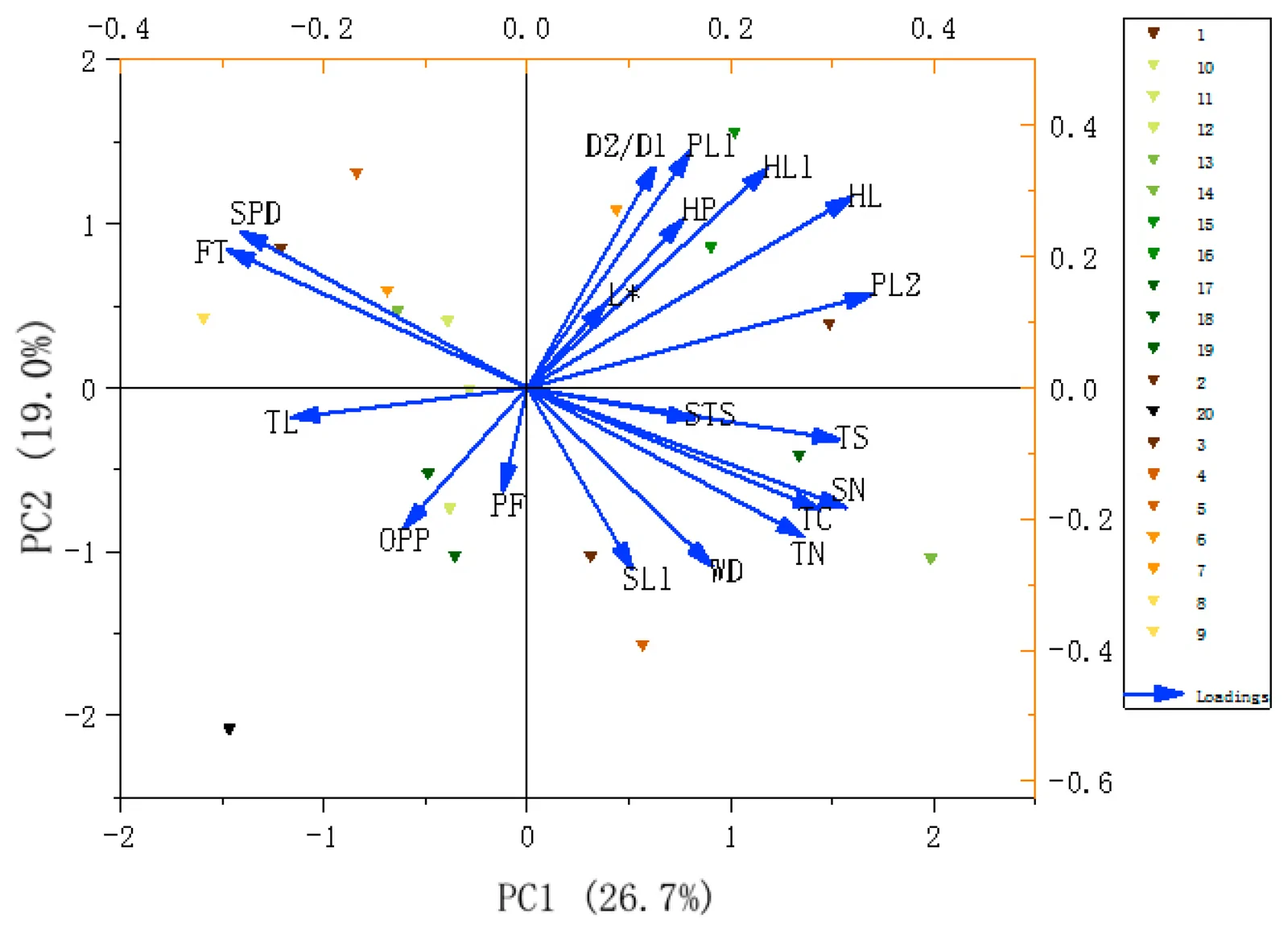
Principal component analysis biplot for floral traits in 20 *H. syriacus* cultivars.

Principal component analysis performed on 20 *H. syriacus* varieties using these 19 indicators yielded five principal components (Table 2, Figure 12) with a cumulative contribution rate of 77.51%. This indicates that the selected components effectively represent the core information regarding fertility-related traits in this species.

Principal Component 1 (PC1: flower size + pollen spine structure factor) accounted for 26.65% of the total variance, representing the most explanatory component. Traits significantly influenced by its eigenvalues included pistil length (PL2), heart length (HL), and stamen number (SN), followed by pollen spine surface microstructure (TS), flower type (FT), and spine curvature (TC). PC2 (pedicel length + floral and pollen proportional structure factor) contributed 19.013%, with primary factors consisting of pedicel length (PL1), the ratio of pollen longitudinal diameters to pollen transverse diameters (D2/D1), and the heart length/petal length ratio (HL1). PC3 (flower lightness + calyx shape + petal posture + heart presence factor) explained 14.22% of the variance, driven by the brightness value (L*), sepal tube shape (STS), outer petal posture (OPP), and heart presence (HP). PC4 (petal shape + pollen spine length factor) contributed 10.07%, with significant traits including petal form (PF) and pollen spine length (TL). Finally, PC5 (stigma–style complex length + heart presence factor) contributed 7.566%, primarily characterized by stigma–style complex length (SL1) and heart presence (HP).

#### 2.4.4. Cluster Analysis

**Figure 13:**
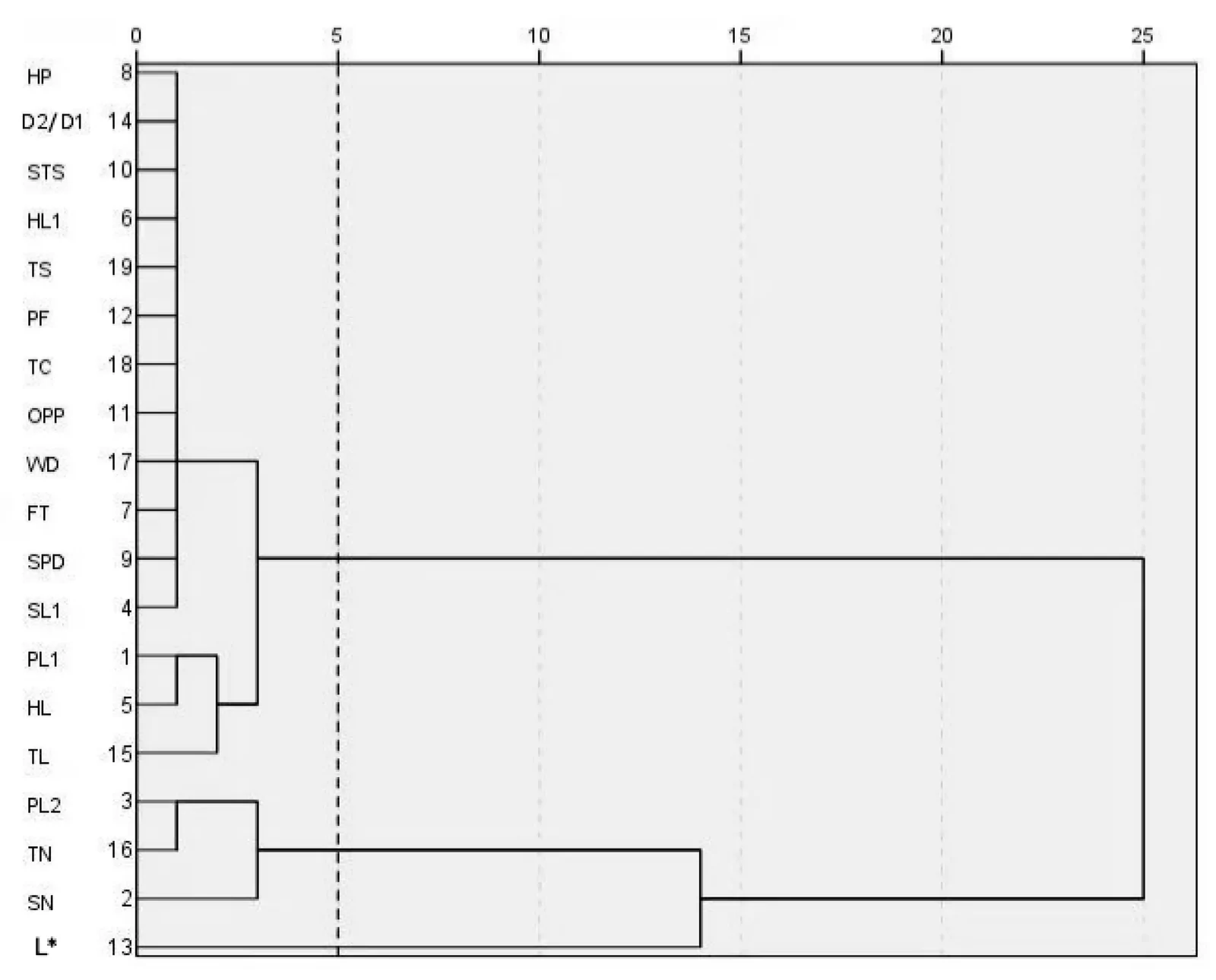
R-type clustering analysis diagram for 19 *H. syriacus* floral traits.

Based on R-type cluster analysis (Figure 13), the 19 traits were categorized into three distinct clusters at a Euclidean distance of 3. Within these clusters, pistil length (PL2) was strongly correlated with pollen spine number (TN), whereas pedicel length (PL1) showed a strong association with heart length (HL). Furthermore, the heart length/petal length ratio (HL1) demonstrated a strong correlation with pollen spine surface (TS) and pollen spine curvature (TC). These seven traits were inferred to be important indicators for distinguishing fertility levels in *H. syriacus*. The scattered distribution of traits shown in Figure 13 indicated that the selection was appropriate for use in subsequent Q-type cluster analysis.

**Figure 14:**
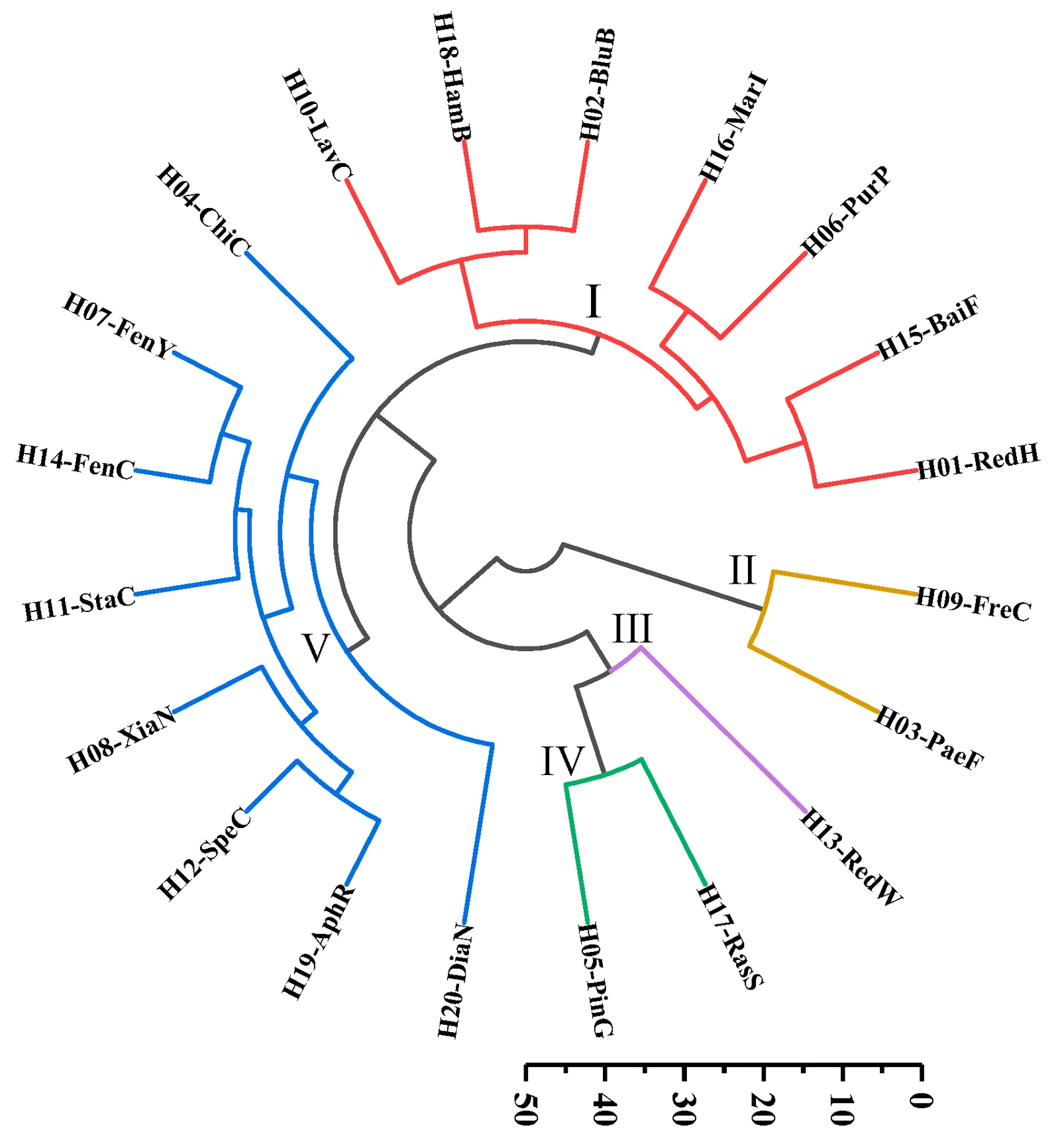
Q-type clustering analysis diagram based on 19 *H. syriacus* floral traits.

Based on the 19 floral traits, Q-type cluster analysis classified the 20 *H. syriacus* cultivars into five distinct groups (Figure 14).

Group I, comprising seven cultivars (35.00%), exhibited high fruit-setting rates. These cultivars were characterized by high values for pedicel length (PL1), pistil length (PL2), stamen number (SN), heart length (HL), and heart length/petal length ratio (HL1), alongside the presence of microspines on the pollen spine surface. Group II (two cultivars, 10.00%) demonstrated low fruit-set rates, double FT, and low pollen spine numbers (TN). Group III (one cultivar, 5.00%) showed a high fruit-set rate and was associated with a significant pistil length (PL2), high stamen number (SN), and high spine number (TN). Group IV (two cultivars, 10.00%) presented low fruit-set rates, spine numbers (TN) exceeding 50, and high rates of pollen abnormality. Finally, Group V (eight cultivars, 40.00%) displayed low fruit-set rates, accompanied by low values for pistil length (PL2), stamen number (SN), and heart length (HL).

## 3. Discussion

### 3.1. Diversity of Floral Traits

Phenotypic diversity serves as the foundation for biodiversity research, and relevant data are widely utilized in the identification, classification, conservation, and genetic utilization of germplasm resources. This approach has been effectively applied to species including *Hibiscus mutabilis* L., *Paeonia suffruticosa* Andr., *Paeonia lactiflora* Pall., *Hibiscus rosa-sinensis* L., and *Gardenia jasminoides* J.Ellis [16,17,18,19,20,21].

Previous investigations by our team confirmed that the floral traits of *H. syriacus* exhibit substantial diversity. Building upon those findings, the current study incorporated floral traits highly relevant to fertility, including pistil length (PL2), stigma color (SC), and ovary shape (OS). At the palynological level, fertility-related indicators such as spine curvature (TC) and pollen spine surface (TS) were also included. When compared with the Guidelines for the Conduct of Tests for Distinctness, Uniformity, and Stability of New Varieties of *H. syriacus* and existing phenotypic indicators for Malvaceae and *H. syriacus*, the parameters selected in this study are more scientifically rigorous. We propose that highly variable floral traits, including style length (PL2) and stamen length (SL), alongside pollen traits, can serve as auxiliary markers for early fertility screening in breeding programs, thereby improving germplasm evaluations in *H. syriacus*. In subsequent phenotypic studies of *H. syriacus*, high-variability traits should be prioritized to facilitate the efficient differentiation of cultivars, while traits with low coefficients of variation, such as calyx length (CL), bract shape (BS), and petal wrinkling degree (PWD), should be avoided.

Among the 20 tested *H. syriacus* cultivars, all quantitative trait diversity indices exceeded 1, reflecting high phenotypic diversity [22]. Traits with high diversity indices included stigma–style complex length (SL1, 1.98), bract length (BL, 1.96), petal index (PI, 1.90), flower color (FC, 1.67), and the degree of overlap between petals (OP, 1.23). The high diversity indices for stigma–style complex length (SL1) and BL suggest that these parameters represent important quantitative floral traits, providing essential morphological evidence of cultivar evolution and pollination adaptation, as well as germplasm classification. Their quantitative trait diversity may facilitate adaptation to pollination requirements across heterogeneous environments, thereby sustaining population reproduction and environmental adaptability. Traits that are not involved in pollination, such as the bract number (BN) and petal wrinkling degree (PWD), possess low diversity indices, which may help to prevent nutrient and energy consumption by non-functional morphological variations.

Traits exhibiting large CV values included calyx width (CW, 52.52%), heart line length (HLL, 51.85%), pedicel length (PL1, 43.88%), sepal tube shape (STS, 75.29%), and stigma color (SC, 75.28%). Specifically, the high CVs for CW and HLL indicate that these traits may be not susceptible to the genetic background but rather to environmental conditions, reflecting strong phenotypic plasticity. We found that, in *H. syriacus*, the average CV for qualitative traits was higher than that for quantitative traits, whereas the diversity index showed the opposite trend. The negative relationship between the diversity index and the CV for the same trait is consistent with findings in *Nymphaea tetragona* Georgi, *Elaeagnus angustifolia* L., and other species [23,24].

The coded quantification of *H. syriacus* quantitative traits can improve cultivar identification [25,26], and a comprehensive set of morphological indicators can establish a more complete classification basis. In this study, principal component and cluster analyses were conducted on a dataset that combined floral phenotypic and pollen morphological traits. The results showed that, in addition to key traits identified in previous research [6], including spine number (TN), pedicel length (PL1), heart length (HL), and the heart length to petal length ratio (HL1), newly added indicators such as pistil length (PL2), pollen spine curvature (TC), and pollen spine surface (TS) serve as important references for these analyses.

Pistil length (PL2) has been widely used as an indicator for principal component and cluster analyses in various plant species, whereas pollen spine curvature (TC) and spine surface (TS) are rarely employed as core analytical indicators. The inclusion of these two traits enriches the quantitative dimensions of pollen morphology. The relatively small sample size analyzed (15 pollen grains per each of the 20 cultivars) may not adequately capture intraspecific variation in pollen morphology, representing a key sampling limitation. Therefore, the available data can only demonstrate correlative relationships. Future research should increase the sample size and incorporate design approaches to further functionally verify the hypothesis that pollen spine curvature (TC) and the presence or absence of small spines on pollen spine surfaces (TS) can improve fertility in *H. syriacus*.

### 3.2. The Significance of Key Traits for Fertility

Fertility in *H. syriacus* is mainly determined by genetic factors, whereas insect pollination patterns, nutrient composition, hormone levels, evolutionary adaptation, and floral phenotypic characteristics exert secondary effects. Although we identified a potential relationship between pollen characteristics and fertility in our previous study [6], we did not undertake a systematic correlation analysis between floral traits and reproductive success. To address this limitation, multiple floral phenotypic and palynological indicators were integrated in this investigation to comprehensively examine their relationships with fertility. Pollen within the Malvaceae family is typically spinose, characterized by either straight or curved spines, with some featuring secondary microspines. Based on these familial traits, pollen spine curvature (TC) and spine surface microspines (TS) were introduced as two targeted and innovative palynological indicators.

Plant morphology primarily regulates fertility through the evolutionary adaptation of pollination modes. Intraspecific trait variation also directly affects fertility. *H. syriacus* flowers have nectaries, large stigmas and approach herkogamy, with stigmas exceeding anthers. Non-coiled stigma lobes and anthers at variable heights on the monadelphous staminal column result in low rates of self-pollination [27]. Though their variability, these floral traits effectively attract insect visitors, which promotes frequent intervariant gene flow and supports the diversity of *H. syriacus* genetic resources.

In this study, we identified five external floral morphological traits that are significantly correlated with fertility in *H. syriacus*: stamen number (SN), pistil length (PL2), heart length (HL), heart line length over heart length (HL2), and the heart length to petal length ratio (HL1). A higher number of stamens enhances the pollen supply and improves fertility. A longer pistil facilitates exogenous pollen adhesion and germination and supports the selection of high-quality male gametes [28]. The association between pistil length and fertility has been widely confirmed in other ornamental species [29,30,31] and crops [32,33]. Regarding heart-related traits, research has shown that *H. rosa-sinensis* individuals with a greater heart length and a higher heart length to petal length ratio attract pollinators, such as honeybees, more effectively [34]. The floral eye pattern serves as a visual signal that increases pollination efficiency and fertility. Other plant species similarly attract pollinators and enhance reproductive success through variations in flower color [35,36], indicating that visual floral traits, such as the *H. syriacus* heart, act as pollination signals and represent a significant pollination regulation strategy.

Pollen morphological characteristics, such as size, shape, and surface structure, directly influence the efficiency of insect-mediated pollen transport and dispersal [37,38]. When insect foraging behavior matches flower morphology, pollen transfer efficiency is significantly improved [39]. In this investigation, we found that four pollen morphological indicators were significantly correlated with fertility: spine length (TL), spine number (TN), spine curvature (TC), and spine surface microspines (TS). Pollen spines seem to enhance adhesion and reduce the risk of pollen being groomed off by insects, thereby benefiting pollination [40]. Here, we found that *H. syriacus* individuals with shorter pollen spines had higher fruit-setting rates, consistent with the preference of honeybees for collecting short-spined Malvaceae pollen [41,42]. Furthermore, only 16.00% of honeybees selected long-spined upland cotton pollen, as long spines hinder dense packing, require additional binding fluid, and increase behavioral costs [43]. Conversely, a higher number of pollen spines increases the probability of deposition and adhesion on the stigma, thereby improving the fruit-setting rate [44]. Flies and butterflies visited *H. syriacus*, but they were not dominant.

Mildly curved spines have a higher adhesion capacity [6]. Lunau et al. (2015) [45] observed that, after the long spines of Alcea rosea pollen were bent, bumblebees began collecting pollen they had previously rejected. The straight, rigid spines found on Malvaceae pollen hinder collection by honeybees, serving as a specific mechanical defense mechanism. However, some *H. syriacus* cultivars (e.g., ‘Pink Giant’, ‘Raspberry Smoothie’) display excessively curved, malformed and shrunken pollen spines, together with poor pollen viability. We hypothesize that pollen malformation in *H. syriacus* is associated with decreased pollen viability, and that these two factors together lead to low fruit-setting rates, as has been explored in *Cucurbita* [46].

We propose that microspines located on pollen spines enhance the probability of pollen adhesion. Comparable secondary microspines and associated particles have been observed on the pollen spines of *Abutilon* and *Gossypium* (Malvaceae), as well as in several plant families [42]. In this study, *H. syriacus* cultivars possessing microspines—specifically, ‘Red Heart’, ‘Red Woodbridge’, and ‘Hamabo’—demonstrated high fruit-setting rates. We suggest that these microspines represent a mechanical adaptation through which plants improve fertility and fruit-set rates. Generally, samples require cleaning and pretreatment for the observation of pollen surface structures (Erdtman, 1952) [47]; nevertheless, germination pores were still detectable via SEM in our study without this treatment, and the variation in the pollen sampling time together with large inter-varietal differences increased the difficulty of observing germination pores. Future research will further explore germination pores and related quantitative indices to deepen our understanding of pollen characteristics. In addition, we found that the degree of pollen wall wrinkling is also associated with fertility, and that this physical trait correlates with specific colorimetric parameters [48,49]. A significant positive correlation was observed between the colorimetric a* value and the degree of pollen wrinkling. We infer that a higher a* value reflects an elevated pigment content, reduced pollen wall stability, and greater wrinkling degree, and vice versa.

## 4. Methods

### 4.1. Experimental Materials

Twenty cultivars of *H. syriacus* L., covering distinct phenotypic traits, were collected from Hunan Botanical Garden (Table 3). These field-grown plants are over five years old and are cared for by professional gardeners with regular irrigation and fertilization. Observations were performed in summer.

### 4.2. Trait Evaluation and Measurement

#### 4.2.1. Measurement of Floral Phenotypic Traits

**Figure 15:**
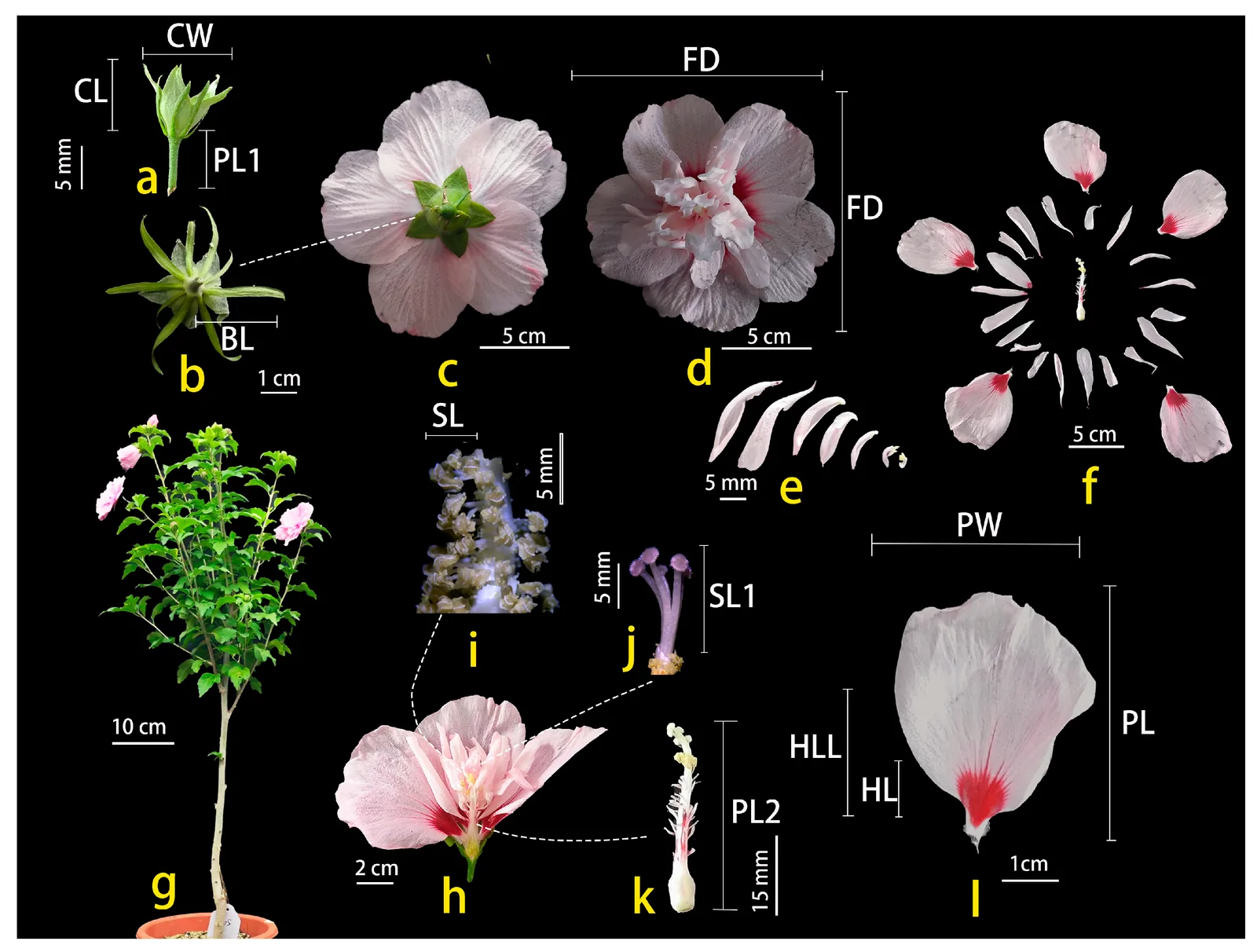
Description of quantitative phenotypic traits in *H. syriacus* flowers. (**a**) Lateral view of sepals and bracts. (**b**) Back view of sepals and bracts. (**c**) Back view of the flower. (**d**) Frontal view of the flower. (**e**) Degenerate stamens. (**f**) Separated floral parts. (**g**) Plant individual. (**h**) Longitudinal section of the flower. (**i**) Group of stamens. (**j**) Stigma–style complex. (**k**) Style. (**l**) Petal.

**Figure 16:**
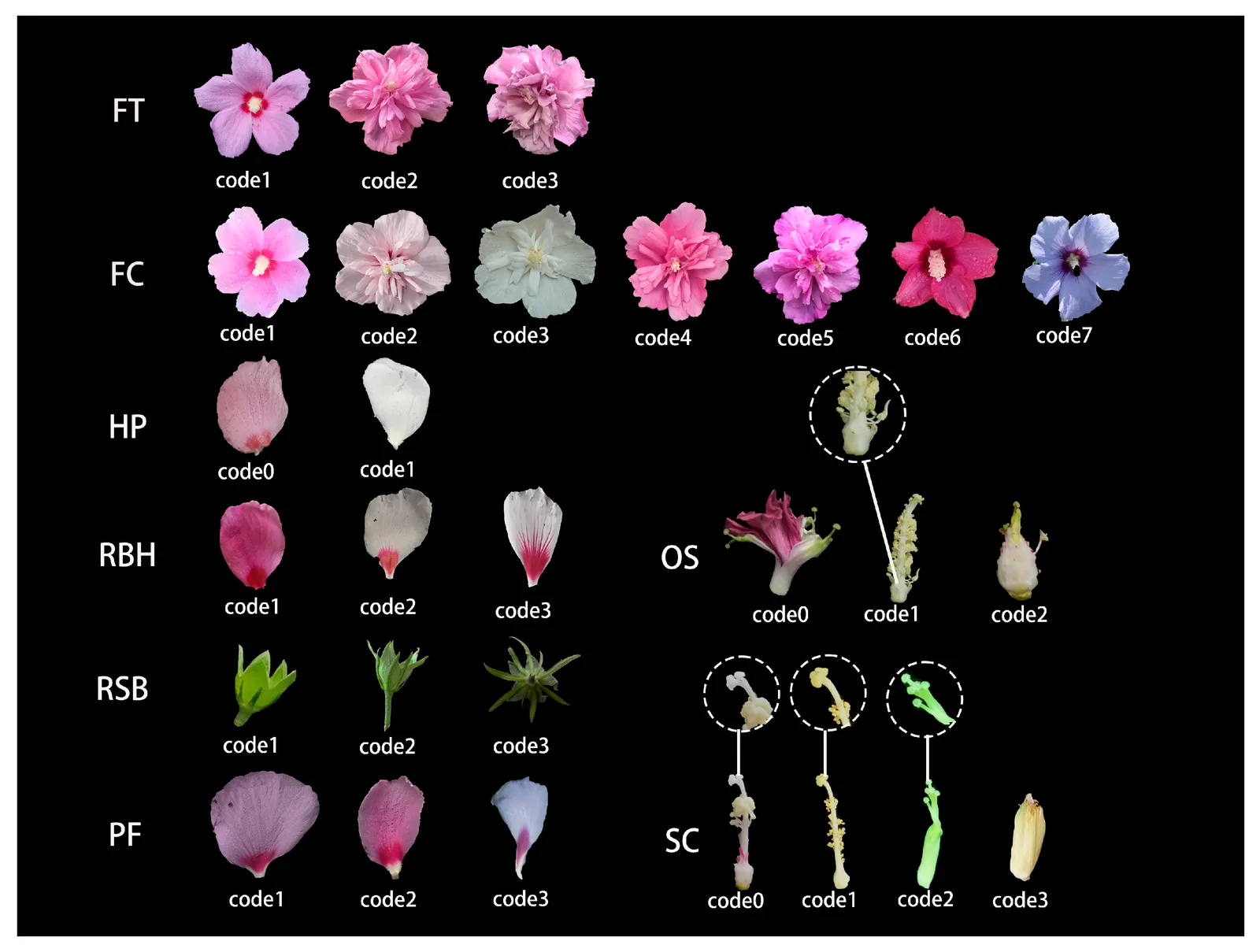
Coded diagram of qualitative phenotypic traits in *H. syriacus* flowers. FT: Petal types are classified into three categories: simple petals, semi-double petals, and double petals; FC: Petal color types include seven variations; HP: Presence or absence of the red heart line; RBH: Relationship between the red heart and the red heart line; RSB: Relationship between sepals and bracts; PF: Petal shapes with different petal indices, including broadly obovate, obovate, and narrowly obovate forms; OS: Ovaries with distinct morphological characteristics; SC: Stigmas with varying colors.

The investigation was performed following the Guidelines for the Conduct of Tests for Distinctness [50], Uniformity and Stability—*Hibiscus syriacus* and *Hibiscus rosa-sinensis* L., and the methodology described by Pan et al. [50,51,52]. Healthy, pest-free and disease-free *H. syriacus* plants were selected for an evaluation of 38 floral traits. Measurements were performed in the morning. Plants were mature field-grown individuals over 5 years old and taller than 1 m. Flowers were sampled on-site, with three measurements per sample and the average value used. These comprised 17 quantitative traits (Figure 15, Table 4), 18 qualitative traits (Figure 16, Table 4), and 3 colorimetric parameters. For the quantitative traits, measurements were taken from 10 flowers at the full-bloom stage, and mean values were calculated. Qualitative traits were assessed through visual assignment (Table S1). Petal color groups were determined using the Royal Horticultural Society Colour Chart (sixth edition, 2015), and color coordinates were subsequently recorded using a KONICA MINOLTA colorimeter (Model: CM-5) [53].

#### 4.2.2. Pollen Morphological Traits

Five randomly selected full-flowering *H. syriacus* flowers from each cultivar were collected, and their pollens were mixed and uniformly mounted on conductive adhesive to prepare samples for electron microscopy, ensuring that the specimens remained intact and normal. The samples were examined and photographed using a JSM 6360-LV electron microscope (JEOL Ltd., Tokyo, Japan), with 12 pollen morphological parameters measured (Table 5). For each parameter, 5 pollen grains were measured per replicate with 3 replicates in total, resulting in 15 pollen grains in all. In total, 12 pollen morphological indicators were measured (Table 5), with mean values derived from 15 randomly selected pollen grains per indicator. These indicators comprised both qualitative and quantitative traits.

### 4.3. Assessment of Fertility Grades and Observation of Insect Flower Visit Characteristics

For the pollen viability assessment, we selected the following materials. A 0.20% benzidine solution, 0.20% α-naphthol solution and 0.25% sodium carbonate solution were prepared and mixed in equal volumes to obtain the staining solution for later use; a 0.30% hydrogen peroxide solution was prepared separately.

Pollen samples were collected on sunny days between 8:00 a.m. and 9:00 a.m. during the full-bloom stage and spread evenly onto glass slides. The staining solution and hydrogen peroxide solution were then added dropwise to the pollen samples, followed by coverslip placement and gentle pressing. After standing for 10 min, the microscopic examination was performed. Three independent visual fields were observed for each sample. Pollen viability was calculated as follows: Pollen viability = (number of viable pollen grains/total number of pollen grains) × 100%. Three biological replicates were analyzed for each cultivar, and the average value was adopted for the experimental results.

The fruit-setting rate serves as the primary indicator of fertility classification [54]. In this study, fruit set was considered successful if the ovary had expanded and remained attached two weeks after pollination.

Field trials were conducted across three consecutive summers from 2023 to 2025, in which twenty *H. syriacus* cultivars were assigned to artificial pollination to verify whether it yields a higher fruit-set rate than open natural pollination and assess its potential to improve fruit setting, with 100 flower buds tagged and labelled for each cultivar.

Artificial pollination group: The petals and stamens were removed from the flower buds. Pollen collected from different plants of the same cultivar was applied to the stigmas between 8:00 and 12:00 on sunny mornings. Immediately after hand pollination, the flowers were enclosed in nylon mesh bags; the bags were removed 2 weeks later.

Open natural pollination group: This group was only tagged, without receiving additional treatments.

Fruit-set rate (%) = (total number of set fruits/total number of tagged flowers) × 100.

Based on three consecutive years of field observations, the fruit-setting rate classification method proposed by Yu Xianghe et al. was adopted in this study [55]. Fertility was classified based on the fruit-setting rate, with a lower limit of >0. The criteria were defined as follows: Grade 1 (0 < fruit-setting rate ≤ 10.00%), Grade 2 (10.00% < fruit-setting rate ≤ 30.00%) and Grade 3 (fruit-setting rate > 30.00%). This classification method, grounded in long-term field data, objectively and accurately reflected the fertility characteristics of *H. syriacus* cultivars.

Observations of insect floral visitors were conducted continuously for 10 days under sunny and breezy conditions across 20 *H. syriacus* cultivars, representing different fertility grades. Daily observations were conducted from 8:00 to 18:00. The observers minimized human interference throughout the whole observation period. The single-plant flower visitation counting method was adopted, whereby observations were carried out on individual plants with abundant blooms. We first counted open flowers on each target plant and recorded pollinator visits from 8:00 to 18:00. The visitation frequency was calculated as: Visitation frequency = Total visits/(Number of open flowers × Observation hours). A valid flower visit was recorded when an insect landed on a flower and then departed. The species and hourly visitation frequency of flower-visiting insects for each cultivar were statistically quantified.

### 4.4. Data Analysis

Statistical analyses were performed using Microsoft Excel 2021. The coefficient of variation (CV) represented the degree of trait dispersion, in which a lower CV indicated greater trait stability. This was calculated using the formula CV (%) = *σ*/*μ* × 100%.

The Shannon–Wiener diversity index (H′) was employed to evaluate phenotypic diversity and was calculated as H′ = −∑*Pi* ln*Pi*. In this equation, *Pi* represents the proportion of cultivars in the *i*-th category of a trait relative to the total number of cultivars, and ln denotes the natural logarithm.

Spearman correlation analysis was performed to assess the relationship between floral traits of *H. syriacus* using SPSS version 25.0. The resulting *p* values were subjected to FDR correction in Excel, with a corrected *q* ≤ 0.1 considered statistically significant [56]. Principal component analysis and hierarchical cluster analysis were conducted on the floral traits of *H. syriacus* using SPSS 25.0 software. The cluster analysis included R-type clustering for traits and Q-type clustering for cultivars. Visualization charts were generated using Origin 2021 software to support the screening of fertility-related traits and the germplasm classification of *H. syriacus*.

## 5. Conclusions

The floral phenotype and pollen morphology of *H. syriacus* exhibit significant diversity. Diversity indices for quantitative traits ranged from 1.23 to 1.98, whereas those for qualitative traits ranged from 0.19 to 1.67. Flower color was categorized into six distinct color groups. Furthermore, the cultivars showed significant differences in pollen grain size, spine morphology and number, surface wrinkle degree (WD), and other morphological indicators.

A total of nine fertility-related indicators were identified, including four pollen morphological indicators (spine length, spine number, spine curvature, and the presence of microspines on the pollen spine surface) and five floral phenotypic indicators (pistil length, heart length, heart length to petal length ratio, heart line length over heart length, and stamen number).

These findings clarify the primary floral phenotypic basis for fruit setting in *H. syriacus* and deepen the current understanding of reproductive regulatory mechanisms. The results also highlight the important contributions to *H. syriacus* fertility of pistil length, pollen spine curvature, and the presence of surface microspines on the pollen spines. The nine screened indicators serve as reliable morphological markers for the rapid screening of hybrid parents. They provide a scientific foundation and key technical support for the genetic improvement of *H. syriacus* and related breeding programs, making a notable contribution to germplasm innovation and the development of high-quality industrial cultivars.

## Figures and Tables

**Table 1:** Statistics of pollination rate and pollen viability.

Cultivar	FRC	Hand-Pollinated Fruit Set (Mean ± SD)	Open-Pollinated Fruit Set (Mean ± SD)	Pollen Vitality (Mean ± SD)
H01-RedH	2	11.85 ± 0.59	10.52 ± 0.53	66.60 ± 2.66
H02-BluB	2	19.89 ± 0.99	17.35 ± 0.87	64.70 ± 2.59
H03-PaeF	1	3.43 ± 0.17	3.21 ± 0.16	13.02 ± 0.52
H04-ChiC	1	1.10 ± 0.06	1.02 ± 0.05	9.06 ± 0.36
H05-PinG	1	4.40 ± 0.22	4.15 ± 0.21	20.65 ± 0.83
H06-PurP	1	8.80 ± 0.44	9.10 ± 0.46	19.80 ± 0.79
H07-FenY	1	1.40 ± 0.07	1.31 ± 0.07	10.50 ± 0.42
H08-XiaN	3	58.00 ± 2.90	44.21 ± 2.21	21.88 ± 0.88
H09-FreC	1	0.50 ± 0.03	0.46 ± 0.02	63.86 ± 2.55
H10-LavC	1	3.50 ± 0.18	4.32 ± 0.22	60.68 ± 2.43
H11-StaC	1	6.10 ± 0.30	8.78 ± 0.44	74.10 ± 2.96
H12-SpeC	1	9.30 ± 0.47	8.75 ± 0.44	69.60 ± 2.78
H13-RedW	3	71.00 ± 3.55	62.80 ± 3.14	20.26 ± 0.81
H14-FenC	1	0.90 ± 0.05	0.44 ± 0.02	23.96 ± 0.96
H15-BaiF	3	75.00 ± 3.75	66.50 ± 3.33	22.58 ± 0.90
H16-MarI	2	18.10 ± 0.91	15.93 ± 0.80	57.56 ± 2.30
H17-RasS	1	0.40 ± 0.02	0.03 ± 0.01	12.94 ± 0.52
H18-HamB	3	81.00 ± 4.05	66.21 ± 3.31	19.14 ± 0.77
H19-AphR	1	2.40 ± 0.12	1.26 ± 0.06	64.60 ± 2.58
H20-DiaN	1	3.80 ± 0.19	2.59 ± 0.13	66.26 ± 2.65

FRC: Fruit retention capacity. 1: 0 < FRC ≤ 10.00%; 2: 10.00% < FRC ≤ 30.00%; 3: FRC > 30.00%.

**Table 2:** Principal component analysis characteristic vector table for 19 fertility-related indicators in *H. syriacus.*

Trait	PC1	PC2	PC3	PC4	PC5
PL1	0.361	0.687	−0.211	−0.067	−0.032
SN	0.706	−0.348	−0.429	−0.042	−0.049
PL2	0.763	0.271	−0.232	0.183	0.309
SL1	0.233	−0.525	0.487	0.149	0.498
HL	0.718	0.553	−0.002	0.277	−0.138
HL1	0.532	0.637	0.225	0.265	−0.177
FT	−0.664	0.401	0.481	0.139	0.213
HP	0.346	0.489	0.502	0.204	−0.483
SPD	−0.635	0.454	0.478	0.033	0.212
STS	0.371	−0.091	0.53	−0.488	0.018
OPP	−0.275	−0.407	−0.527	0.143	−0.404
PF	−0.054	−0.299	0.337	−0.753	−0.2
L*	0.175	0.245	−0.63	−0.479	0.248
D2/D1	0.285	0.65	−0.239	−0.42	0.295
TL	−0.522	−0.087	−0.393	0.513	0.3
TN	0.611	−0.43	0.265	0.041	0.251
WD	0.409	−0.516	0.162	0.361	−0.136
TC	0.647	−0.353	0.15	−0.038	−0.207
TS	0.691	−0.151	0.144	0.123	0.387
E	5.064	3.613	2.701	1.913	1.438
CR%	26.651	19.013	14.217	10.066	7.566
CCR%	26.651	45.664	59.881	69.948	77.514

E: Eigenvalues; CR: Contribution rate; CCR: Cumulative contribution rate.

**Table 3:** List of tested cultivars.

No.	Cultivar	Origin	No.	Cultivar	Origin
H01-RedH	Red Heart	CN	H11-StaC	Starlight Chiffon	UK
H02-BluB	Blue Bird	USA	H12-SpeC	Wild-type Species	CN
H03-PaeF	Paeonyflorus	CN	H13-RedW	Red Woodbridge	KR
H04-ChiC	China Chiffon	CN	H14-FenC	Fen Chiffon	UK
H05-PinG	Pink Giant	KR	H15-BaiF	Bai Fengche	CN
H06-PurP	Purple Pillar	ES	H16-MarI	Marina	USA
H07-FenY	Fen Yun	CN	H17-RasS	Raspberry Smoothie	USA
H08-XiaN	Xiao Nvhai	CN	H18-HamB	Hamabo	JP
H09-FreC	French Cabaret Purple	FR	H19-AphR	Aphrodite	USA
H10-LavC	Lavender Chiffon	UK	H20-DiaN	Diana	USA

CN: China; USA: United States of America; JP: Japan; UK: United Kingdom; FR: France; ES: Spain; KR: Korea.

**Table 4:** Measurement parameters of phenotypic traits in *H. syriacus* flower parts.

NO	Character	Code	NO	Character	Code
N-1	Flower diameter	FD	Q-1	Flower type	FT
N-2	Petal length	PL	Q-2	Flower color	FC
N-3	Petal width	PW	Q-3	Heart presence	HP
N-4	Petal index (PW/PL × 100)	PI	Q-4	Stamen petalody degree	SPD
N-5	Pedicel length	PL1	Q-5	Stigma color	SC
N-6	Stamen number	SN	Q-6	Relationship between heart and heart line Heart line	RBH
N-7	Stamen length	SL	Q-7	Heart base shape	HBS
N-8	Pistil length	PL2	Q-8	Sepal tube shape	STS
N-9	Stigma–style complex length	SL1	Q-9	Sepal shape	SS
N-10	Heart length	HL	Q-10	Bract shape	BS
N-11	Heart line length	HLL	Q-11	Relationship between sepal and bract	RSB
N-12	Heart length over petal length ratio	HL1	Q-12	Outer petals posture	OPP
N-13	Heart line length over heart length ratio	HL2	Q-13	Petal notch	PN
N-14	Calyx length	CL	Q-14	Overlap between petals	OP
N-15	Calyx width	CW	Q-15	Petal form	PF
N-16	Bract number	BN	Q-16	Petal wrinkling degree	PWD
N-17	Bract length	BL	Q-17	Flower color number	FCN
			Q-18	Ovary shape	OS

N: Quantitative traits; Q: Quality traits. Petal index: Petal width/Petal length × 100.

**Table 5:** Pollen morphological indicators.

Character	Code	Type	Grading Criteria
Pollen grain shape	PGS	Q	Nearly spherical, 0; spherical, 1
Transverse diameters	D1	N	/
Longitudinal diameter	D2	N	/
Longitudinal diameter/Transverse diameters	D2/D1	N	/
Spine length	TL	N	/
Spine number	TN	N	/
Spine distance	TD	N	/
Spine base width	TBW	N	/
Spine length/Spine base width	TL/TBW	N	/
Wrinkle degree	WD	Q	Smooth, 0; mild, 1; moderate, 2; serious, 3
Spine curvature	TC	Q	Straight, 1; slightly curved, 2
Pollen spine surface	TS	Q	Smooth, 0; microspinous, 1

Note: N: Quantitative traits; Q: Quality traits.

## Data Availability

The datasets generated and analyzed during this study are available from the corresponding author on reasonable request. Plant material collection complied with local regulations and institutional guidelines.

## Supplementary Materials

The following supporting information can be downloaded at: https://www.mdpi.com/article/10.3390/plants15213492/s1, Figure S1: Pollen population view (×100); Figure S2: Pollen spine structure; Table S1: Coding of quality traits of *H. syriacus*; Table S2: Diversity analysis of quantitative traits in *H. syriacus* cultivars; Table S3: Diversity analysis of quality traits in *H. syriacus* cultivars; Table S4: Measurement of 12 pollen traits in *H. syriacus* cultivars; Table S5: Correlation analysis and FDR correction of *p* values; Table S6: correlation analysis between pollen traits and floral phenotypic traits.

## Abbreviations

Abbreviation	Meaning
TLA	Three-letter acronym
FD	Flower diameter
PL	Petal length
PW	Petal width
PI	Petal index
PL1	Pedicel length
SN	Stamen number
SL	Stamen length
PL2	Pistil length
SL1	Stigma–style complex length
HL	Heart length
HLL	Heart line length
HL1	Heart length over petal length ratio
HL2	Heart line length over heart length ratio
CL	Calyx length
CW	Calyx width
BN	Bract number
BL	Bract length
FT	Flower type
FC	Flower color
HP	Heart presence
SPD	Stamen petalody degree
SC	Stigma color
RBH	Relationship between heart and heart line
HBS	Heart base shape
STS	Sepal tube shape
SS	Sepal shape
BS	Bract shape
RSB	Relationship between sepal and bract
OPP	Outer petals posture
PN	Petal notch
OP	Overlap between petals
PF	Petal form
PWD	Petal wrinkling degree
FCN	Flower color number
OS	Ovary shape
PGS	Pollen grain shape
D1	Pollen transverse diameters
D2	Pollen longitudinal diameters
D2/D1	Pollen longitudinal diameters/Pollen transverse diameters
TL	Spine length
TN	Spine number
TD	Spine distance
TBW	Spine base width
TL/TBW	Spine length/Spine base width
WD	Wrinkle degree
TC	Spine curvature
TS	Pollen spine surface
