# Supplementary material for: Analysis of Floral Trait Diversity and Its Correlation with Fertility in Hibiscus syriacus Cultivars
Source: Plants (Basel). 2026 Nov 9;15(21):3492. doi: 10.3390/plants15213492 (PMC13766213; doi:10.3390/plants15213492)
Supplement: Supplementary file 1 [file plants-15-03492-s001.zip › plants-4615006-supplementary.pdf]

The supplementary figures and tables

Table S1 Coding of quality traits of *Hibiscus syriacus*
Table S2 Diversity analysis of quantitative traits in *Hibiscus syriacus* cultivars
Table S3 Diversity analysis of quality traits in *Hibiscus syriacus* cultivars
Table S4 Measurement of 12 pollen traits in *Hibiscus syriacus* cultivars
Table S5 Correlation analysis and FDR correction of P values
Table S6 Correlation analysis between pollen traits and floral phenotypic traits

Figure S1 Pollen population view (×100)
Figure S2 Pollen spine structure

.

Table S1 . Coding of quality traits of *Hibiscus syriacus*

Character	Code	Grading criteria							
		0	1	2	3	4	5	6	7
phenotypic									
Flower type	FT		single petal	semi-double	double				
Flower colour	FC		pinkish purple	pinkish white	white	pink	purple	reddish purple	bluish purple
Heart presence	HP	none	present						
Stamen petalody degree	SPD		no	mild	moderate	high			
Stigma color	SC	white	yellow	green	no stigma				
Relationship between heart and heart line	RBH		nearly equal length	exceeding	exceeding significantly				
Heart base shape	HBS	truncate	wedge-shaped	none					
Sepal tube shape	STS	bell-shaped	bowl-shaped						
Sepal shape	SS		triangular	elongated triangular					

Bract shape	BS	needle-shaped	linear	lanceolate			
Relationship between sepal and bract	RSB		calyx below bract	close to	calyx above bract		
Outer petals posture	OPP		upward	spreading			
Petal notch	PN		none or very weak	moderate	strong		
Overlap between petals	OP		mild overlap	moderate overlap	high overlap	mild separation	high separation
Petal form	PF		broadly obovate	obovate	narrowly obovate		
Petal wrinkling degree	PWD		none or weak	moderate	strong		
Flower colors number	FCN		monochromatic	polychromatic			
Ovary shape	OS	no ovary	suitable	enlarged			
Pollen							
Pollen grain shape	PGS	Nearly spherical	Spherical				

Wrinkle degree	WD	Smooth	Mild	moderate	serious
Thorn curvature	TC		Straight	Slightly curved	
Pollen thorn surface	TS	Smooth	Microspinose		

Table S2.Diversity analysis of quantitative traits in *Hibiscus syriacus* cultivars

Code	maximum	minimum	σ	u	Cv(%)	H'
FD	103.69	48.43	19.14	74.73	25.61	1.85
PL	77.01	35.11	10.19	54.50	18.69	1.94
PW	66.88	21.81	11.03	39.52	27.90	1.92
PI	99.38	51.40	12.02	72.05	16.68	1.90
PL1	19.66	4.04	4.28	9.75	43.88	1.71
SN	55.00	0	14.54	34.95	41.60	1.45
SL	6.60	0	1.45	3.76	38.59	1.73
PL2	50.42	32.22	6.03	39.94	15.09	1.78
SL1	11.11	3.67	1.86	6.74	27.64	1.98
HL	21.42	0	4.15	11.78	35.22	1.39
HLL	45.87	0	9.14	17.62	51.85	1.61
HL1	0.36	0	0.07	0.22	33.77	1.53
HL2	2.44	0	0.51	1.40	36.05	1.36
CL	24.98	11.57	3.79	19.58	19.38	1.58
CW	44.58	6.96	8.18	15.55	52.62	1.52
BN	9.00	6.00	0.92	7.00	13.11	1.23
BL	24.00	7.00	4.53	15.18	29.83	1.96
mean					31.03	1.67

Table S3. Diversity analysis of quality traits in *Hibiscus syriacus* cultivars

	Distribution frequencies								Cv. (%)	H'
Code	0	1	2	3	4	5	6	7		
FT	0	0.6	0.2	0.2	0	0	0	0	51.299	0.95
FC	0	0.05	0.2	0.2	0	0.3	0.15	0.1	44.414	1.67
HP	0.05	0.95	0	0	0	0	0	0	23.538	0.199

SPD	0	0.55	0.1	0.2	0.15	0	0	0	61.076	1.166
SC	0.35	0.65	0	0	0	0	0	0	75.286	0.647
RBH	0.3	0.6	0.1	0	0	0	0	0	34.199	0.898
HBS	0.2	0.75	0.05	0	0	0	0	0	57.572	0.687
STS	0.35	0.65	0	0	0	0	0	0	75.285	0.647
SS	0	0.55	0.45	0	0	0	0	0	35.201	0.688
BS	0.55	0.45	0	0	0	0	0	0	34.199	0.688
RSB	0	0.55	0.4	0.05	0	0	0	0	40.465	0.845
OPP	0	0.6	0.4	0	0	0	0	0	35.902	0.673
PN	0	0.75	0.25	0	0	0	0	0	35.541	0.562
OP	0	0.5	0.3	0.05	0.1	0.05	0	0	63.664	1.238
PF	0	0.2	0.8	0	0	0	0	0	22.8	0.5
PWD	0	0.85	0.15	0	0	0	0	0	31.856	0.423
FCN	0	0.9	0.1	0	0	0	0	0	35.13	0.325
OS	0	0.9	0.1	0	0	0	0	0	27.981	0.325
mean									43.633	0.729

Table S4. Measurement of 12 pollen traits in *Hibiscus syriacus* cultivars

cultivated varieties	PGS	WD	TC	TS	D1	D2	D2/D1	TL	TN	TD	TBW	TL/TBW
Red Heart	1	1	2	1	119.33	119.33	1	18.47	38	21.96	8.71	2.12
Blue Bird	0	2	2	0	134.67	129.2	0.96	16.74	34	22.29	9.99	1.68
Paeonyflorus	0	2	1	0	129.35	127.68	0.99	24.43	30	26.62	10.68	2.29
China Chiffon	0	1	1	0	130.28	128.73	0.99	18.61	28	31.04	9	2.07
Pink Giant	0	3	2	0	154.75	148.82	0.96	19.07	45	20.07	7.81	2.44
Purple Pillar	0	1	1	0	129.22	126.87	0.98	21.55	30	24.56	9.77	2.21
Fen Yun	1	1	1	0	115.52	115.52	1.00	17.77	30	23.63	8.7	2.04
Xiao Nvhai	1	1	2	0	118.22	118.22	1.00	15.86	36	22.40	8.67	1.83
French Cabaret Purple	0	0	1	0	118.76	116.2	0.98	22.33	29	24.53	10.92	2.04

Lavender Chiffon	0	1	1	0	145.86	144.07	0.99	22.91	40	23.89	10.31	2.22
Starlight Chiffon	0	0	1	0	117.12	116.69	0.99	18.14	35	19.17	9.96	1.82
Species	0	2	2	0	122.73	119.72	0.98	22.48	30	23.63	9.46	2.38
Red Woodbridge	0	3	2	1	151.76	149.74	0.99	17.26	58	18.24	8.15	2.12
Fen Chiffon	1	0	1	0	124.34	124.34	1.00	16.95	32	20.63	9.12	1.86
Bai Fengche	0	0	2	0	130.89	136.4	1.04	20.31	40	26.55	9.20	2.21
Marina	0	2	1	0	124.26	127.76	1.03	15.39	37	26.48	8.68	1.77
Raspberry Smoothie	0	1	2	0	142.99	137.87	0.96	16.90	50	17.50	8.23	2.05
Hamabo	0	2	2	1	131.20	129.93	0.99	17.81	38	23.74	9.89	1.80
Aphrodite	0	2	1	0	120.76	119.22	0.99	19.95	29	23.40	9.44	2.11
Diana	0	1	1	0	125.98	123.86	0.98	24.75	35	22.03	10.07	2.46
mean	0.2	1.3	1.45	0.15	129.40	128.01	0.99	19.38	36.2	23.12	9.34	2.08
standard deviation	0.41	0.92	0.51	0.37	11.47	10.54	0.02	2.82	7.71	3.19	0.86	0.23
minimum					115.52	115.52	0.96	15.39	28.0	17.50	7.81	1.68
maximum					154.75	149.74	1.04	24.75	58.0	31.04	10.92	2.46
range					39.23	34.22	0.08	9.36	30.0	13.54	3.11	0.78
coefficient of variation					8.86	8.23	2.00	14.54	21.3	13.82	9.18	10.93

Table S5. Correlation analysis and FDR correction of P values

quantitative floral traits	Correlation Coefficient	raw P-values	Rank	P-FDR	BH-sig
**HL**	.630**	0.003	1	**0.05**	**yes**
**HL1**	.579**	0.007	2	**0.06**	**yes**
**HL2**	-.549*	0.012	3	**0.07**	**yes**
**PL2**	.509*	0.022	4	**0.09**	**yes**
**SN**	.475*	0.034	5	**0.10**	**yes**
BN	0.438	0.053	6	0.15	no
PL1	0.283	0.226	7	0.55	no
SL	0.279	0.233	8	0.50	no
BL	0.278	0.235	9	0.44	no
*L**	0.274	0.243	10	0.41	no
HLL	0.269	0.251	11	0.39	no
CL	-0.229	0.331	12	0.47	no
*b**	0.172	0.467	13	0.61	no

PL	0.138	0.562	14	0.68	no
FD	0.134	0.573	15	0.65	no
PI	-0.076	0.752	16	0.80	no
CW	-0.06	0.803	17	0.80	no
SL1	0.058	0.809	18	0.76	no
PW	0.02	0.932	19	0.83	no
*a**	0.006	0.979	20	0.83	no
qualitative floral traits	Correlation Coefficient	raw P-values	Rank	P-FDR	
FT	-.567	0.009	1	0.16	no
OP	-.498	0.026	2	0.23	no
SPD	-.471	0.036	3	0.22	no
OPP	-0.418	0.066	4	0.30	no
SC	0.355	0.125	5	0.45	no
RSB	0.339	0.144	6	0.43	no
STS	0.279	0.233	7	0.60	no
SS	-0.247	0.293	8	0.66	no
OS	-0.239	0.31	9	0.62	no
FC	0.187	0.43	10	0.77	no
HP	0.165	0.488	11	0.80	no
PF	0.154	0.518	12	0.78	no
FCN	0.154	0.518	13	0.72	no
PN	-0.142	0.55	14	0.71	no
RBD	-0.072	0.761	15	0.91	no
PWD	-0.072	0.764	16	0.86	no
HBS	0.068	0.775	17	0.82	no
BS	-0.064	0.788	18	0.79	no
pollen traits	Correlation Coefficient	raw P-values	Rank	P-FDR	
**TC**	.628**	0.003	1	**0.04**	**yes**
**TS**	.588**	0.006	2	**0.04**	**yes**
**TN**	.503*	0.024	3	**0.10**	**yes**
**TL**	-.494*	0.027	4	**0.08**	**yes**
D2/D1	0.41	0.073	5	0.18	no
TL/TBW	-0.363	0.115	6	0.23	no
TBW	-0.295	0.207	7	0.35	no
D2	0.273	0.244	8	0.37	no
D1	0.148	0.532	9	0.71	no
PGS	0.141	0.553	10	0.66	no
WD	0.124	0.602	11	0.66	no
TD	-0.007	0.976	12	0.98	no

Note:BH-sig.(P≤0.1): Yes = significant, No = non-significant. *: P<0.05, **: P<0.01. Bold traits have significant correlations with fertility

Table S6.Correlation analysis between pollen traits and floral phenotypic traits

Floral phenotype		Pollen Traits											
		PGS	D1	D2	D2/D1	**TL**	**TN**	TD	TBW	TL/TBW	WD	**TC**	**TS**
FD		0.043	0.125	0.212	0.261	-0.101	0.373	0.008	0.078	-0.077	-0.139	-0.026	0.085
PL		0	0.281	0.346	0.263	0.087	0.416	-0.186	-0.269	0.325	0.033	0.009	0.231
PW		-0.13	0.241	0.32	0.276	0.126	0.294	-0.011	-0.122	0.292	-0.004	-0.183	0.109
PI		-0.195	0.245	0.319	0.165	0.065	0.221	0.168	-0.035	0.164	0.01	-0.2	-0.061
PL1		-0.108	0.033	0.171	0.263	-0.155	0.092	0.385	-0.104	-0.11	-0.177	0.078	-0.085
**SN**		-0.164	.477*	.494*	-0.044	-0.031	.551*	-0.233	-0.005	0.088	0.214	0.43	.476*
SL		-0.26	.460*	.508*	-0.042	-0.229	0.235	0.143	-0.319	-0.062	0.415	0.253	0.279
**PL2**	Quantitative traits	-0.108	0.338	.496*	0.295	-0.107	.707**	-0.053	-0.314	0.137	0.112	0.357	.498*
SL1		-0.195	0.392	0.359	-0.3	-0.084	.457*	-0.35	-0.123	0.011	0.406	0.148	0.425
**HL**		0	0.177	0.29	.466*	-0.14	0.231	0.338	-0.209	-0.087	-0.007	0.2	.474*
HLL		0.043	-0.023	0.078	.498*	-0.015	0.075	0.308	0.056	-0.143	-.466*	-0.166	0.182
**HL1**		0.108	-0.009	0.066	0.362	-0.162	0.021	0.443	-0.132	-0.2	-0.098	0.2	0.401
**HL2**		0.022	-0.268	-0.292	0.011	0.223	-0.433	0.267	0.317	-0.038	-.542*	-.514*	-.546*
CL		0.043	0.054	0.12	0.337	0.054	0.059	0.093	-0.319	0.176	-0.107	-0.34	-0.134
CW		-0.087	-0.095	-0.053	-0.032	-0.242	-0.035	0.13	0.002	-0.389	-0.183	-0.218	-0.036

BN		-0.137	0.439	.461*	0.116	-0.234	0.184	0.285	-0.038	-0.215	0.192	0.138	0.166
BL		-0.13	0.332	0.402	0.272	-0.162	0.181	0.059	-0.129	-0.116	-0.034	-0.061	0.158
FT		0.099	-0.192	-0.24	0.031	0.144	-0.403	0.223	0.267	-0.096	-0.336	-.556*	-0.332
FC		-0.333	0.315	0.339	-.446*	-0.332	0.381	-0.319	-0.312	-0.105	.498*	0.348	-0.087
HP		0.115	0.02	0.099	0.14	-0.378	0	0.139	-0.259	-0.378	0.104	0.208	0.096
SPD		0.024	-0.14	-0.167	0.09	0.163	-0.342	0.265	0.268	-0.069	-0.428	-.471*	-0.361
SC		-0.157	0.027	0.082	0.192	-0.118	0.055	0.109	0.245	-0.3	-0.331	0.032	0.015
RBD		-0.025	-0.345	-0.299	0.131	-0.119	-0.358	0.178	0.116	-0.316	-.456*	-0.36	-0.335
HBS		0.172	0.087	0.017	-0.037	-0.038	0.085	-0.349	-0.142	0.113	0.008	0.092	0.145
STS		0.105	-0.009	0.045	0.109	-.518*	0.311	-0.418	-0.318	-.482*	0.085	0.242	0.308
SS		0.05	-0.131	-0.078	0.009	-0.253	-0.079	0.105	-0.357	-0.131	0.127	-0.212	-0.38
BS	Quality traits	-0.25	0.022	0	0.152	0.347	-0.251	0.282	0.087	0.303	-0.203	-0.201	-0.21
RSB		0.025	-0.08	-0.095	0.163	-0.131	-0.069	0.054	0.278	-0.325	-0.188	0.04	0.152
OPP		-0.408	0.053	-0.035	-.515*	0.265	-0.089	-0.239	0.372	0.159	0.092	-0.123	-0.343
PN		0	-0.03	-0.01	0.181	-0.07	-0.161	0.16	-0.29	-0.01	-0.01	-0.058	-0.243
OP		-0.282	-0.204	-0.239	-0.333	0.353	-0.331	0.182	0.161	0.215	-0.212	-0.151	-0.395
PF		-0.062	-0.087	-0.065	-0.054	-0.434	0	-0.282	-0.325	-0.39	0.079	0.201	-0.14
PWD		-0.21	0.036	-0.061	-0.329	-0.036	-.464*	0.279	0.304	-0.304	-0.164	-0.099	-0.176
FCN		0.25	-0.145	-0.145	0.276	-0.173	-0.029	0.101	-0.029	-0.318	0.045	0.034	0.327
OS		0.25	-0.231	-0.289	0.015	0	-0.305	-0.029	0.231	-0.202	-.482*	-0.302	-0.14

L*		0.303	-0.266	-0.194	.525*	-0.086	-0.01	0.025	0.081	-0.149	-.497*	-0.009	0.182
a*	Colorimeter	-0.238	0.395	0.364	-0.372	0.083	0.278	-0.184	-0.155	0.278	.567**	0.253	0.036
b*	Parameters	0.13	-0.063	-0.03	0.414	-0.026	-0.067	0.217	0.09	-0.168	-0.432	-0.078	0.158

Note:*p< 0.05 ;**p<0.01. Bold English codes indicate indicators significantly correlated with fertility

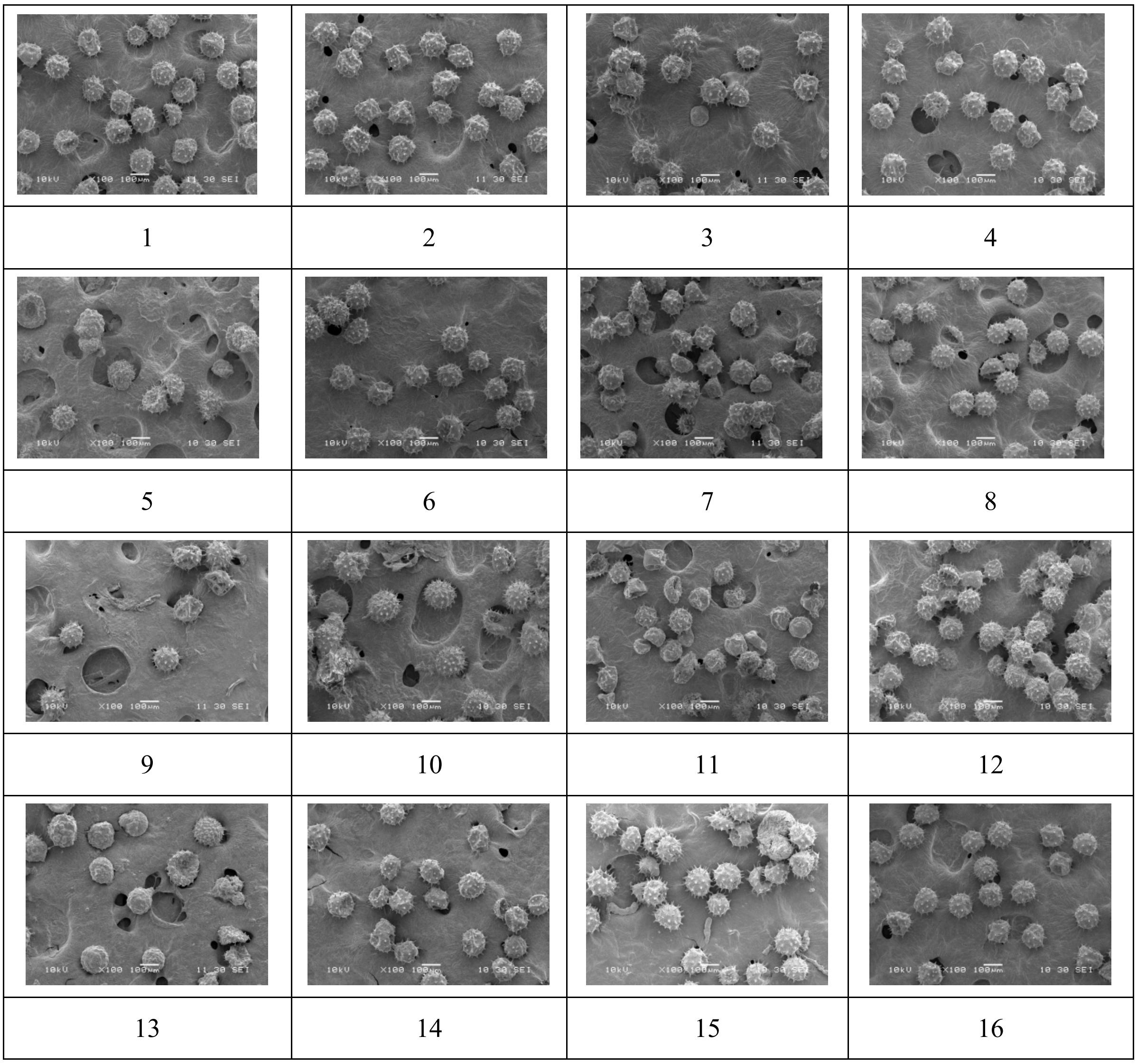

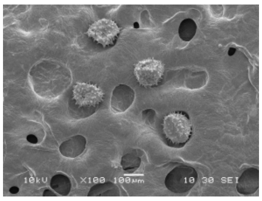	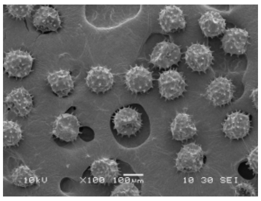	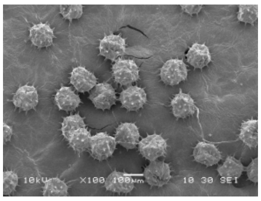	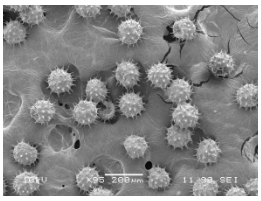
17	18	19	20

Figure S1.Pollen population view (×100)

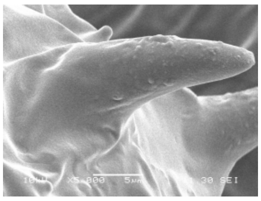	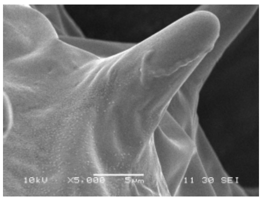	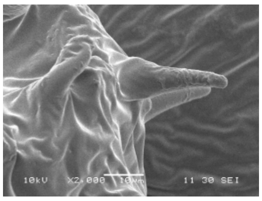	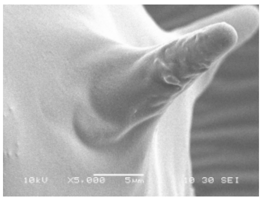
1	2	3	4
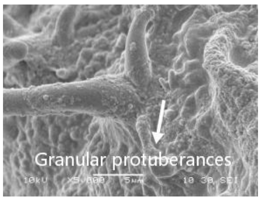	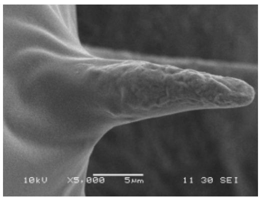	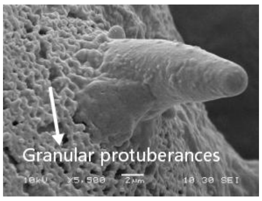	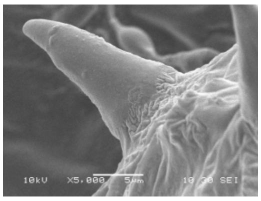
5	6	7	8
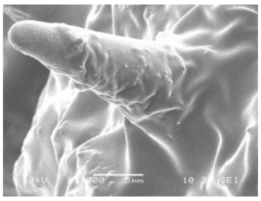	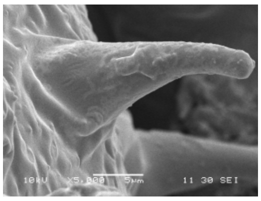	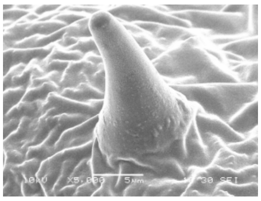	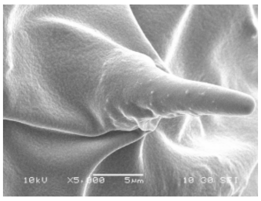
9	10	11	12
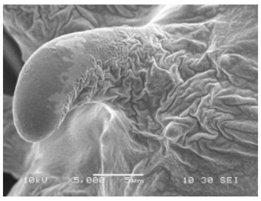	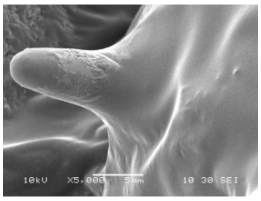	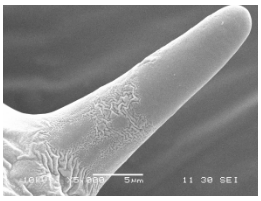	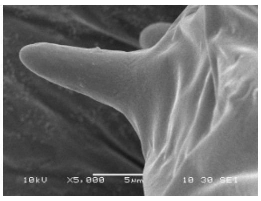
13	14	15	16

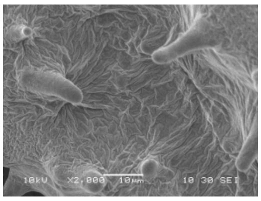	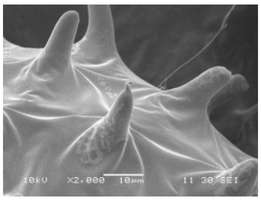	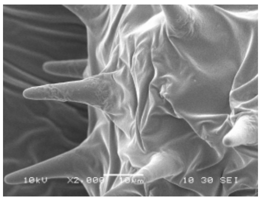	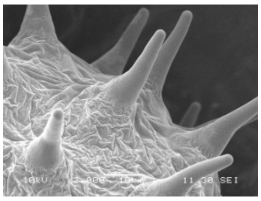
17	18	19	20

Figure S2.Pollen spine structure
